# A Biomarker‐Based Dose–Schedule Optimization Design for Immunotherapy Trials

**DOI:** 10.1002/sim.70357

**Published:** 2026-01-22

**Authors:** Yingjie Qiu, Yan Han, Beibei Guo

**Affiliations:** ^1^ Peter O'Donnell Jr. School of Public Health University of Texas Southwestern Medical Center Dallas Texas USA; ^2^ Harold C. Simmons Comprehensive Cancer Center University of Texas Southwestern Medical Center Dallas Texas USA; ^3^ Department of Biostatistics and Health Data Science Indiana University Indianapolis Indiana USA; ^4^ Department of Experimental Statistics Louisiana State University Baton Rouge Louisiana USA

**Keywords:** Bayesian adaptive design, biomarker, immunotherapy, optimal treatment regime, phase I/II trial, risk‐benefit tradeoff, subgroups

## Abstract

In immunotherapy, both the dose and the schedule of drug administration can significantly influence therapeutic effects by modulating immune system activation. Incorporating immune response measures into clinical trial designs offers an opportunity to enhance decision‐making by leveraging their close association with therapeutic efficacy and toxicity. Motivated by settings where biomarker data indicate improved efficacy in biomarker‐positive patients, we propose a dose–schedule optimization strategy tailored to each biomarker‐defined subgroup, based on elicited utility functions that capture risk‐benefit tradeoffs. We introduce a joint modeling framework that simultaneously evaluates immune response, toxicity, and efficacy, enabling information sharing across outcome types and patient subgroups. Our approach utilizes parsimonious yet flexible models designed specifically to address challenges due to small sample sizes commonly encountered in early‐phase trials. Simulation studies demonstrate that the proposed design achieves desirable operating characteristics and effectively informs dose–schedule optimization.

## 1 Introduction

Immunotherapy has achieved unprecedented success in the treatment of various cancers such as melanoma, non‐small cell lung cancer, renal cell carcinoma, hepatocellular carcinoma, and esophageal cancer. A key feature of immunotherapy is its mechanism of action‐stimulating the patient's immune system to target and eliminate cancer cells. Accordingly, the immune response serves as a critical outcome of biological activity, providing insight into the therapy's ability to activate the immune system. Given the close association between immune response and both therapeutic efficacy and toxicity [1, 2, 3], it is essential to incorporate immune response into trial designs to support more informed and efficient decision‐making. Various dose‐finding designs for immunotherapy have been developed that can incorporate an immune response as a component of decision rules [4, 5, 6, 7, 8, 9].

All the above immunotherapy trial designs assume patient homogeneity and aim to identify a single optimal dose for the entire patient population. However, emerging evidence has revealed that various factors may affect immunotherapy effectiveness, including predictive biomarkers [10, 11, 12, 13, 14, 15, 16, 17], gender [18], age [19, 20], and obesity [21, 22]. In particular, several biomarkers reflecting immune activation have been shown to influence therapeutic response. For example, PD‐L1 expression levels have been prospectively used to guide pembrolizumab therapy in KEYNOTE‐001 [12] and KEYNOTE‐010 [16]. Moreover, tumor mutational burden [15] and interferon‐γ‐related mRNA signatures, which represent cytokine‐driven immune activation [17], have been associated with heterogeneous response patterns across checkpoint inhibitors. These findings imply that the optimal dose of immunotherapy may differ across patient subgroups. To address this heterogeneity, Guo and Zang [23, 24] have recently proposed phase I/II designs for immunotherapy that aim to determine the optimal dose of immunotherapy in each patient subgroup.

In practice, immunotherapy is typically administered according to a prespecified treatment schedule, which outlines the number of treatment cycles and the timing and frequency of therapy. Numerous studies have investigated the impact of treatment schedule on immunotherapy efficacy and toxicity [25, 26, 27, 28]. Nevertheless, as noted by Lesterhuis et al. [29], finding the optimal biological dose and schedule for immunotherapy is much more challenging than for traditional chemotherapy, and there is an urgent need for clinical trial designs to determine the best dose and schedule for immunotherapies. Unfortunately, the existing body of literature has largely neglected the impact of treatment schedule on the effectiveness of immunotherapy by assuming a fixed schedule.

In this article, we develop a Bayesian biomarker‐based phase I/II trial design to optimize subgroup‐specific dose–schedule combinations for immunotherapy trials. In the design, we simultaneously consider the three endpoints, including immune response, toxicity, and efficacy. We use a plateau model for immune response, a logistic model for toxicity, and flexible Bayesian dynamic models to evaluate efficacy, enabling information sharing among dose–schedule combinations. Considering the relatively small sample size in a typical phase I/II trial, the models in the proposed design are parsimonious yet flexible to facilitate information borrowing across dose–schedule combinations and subgroups. We quantify each dose–schedule combination's desirability using a utility function that reflects the desirability tradeoffs between toxicity and efficacy. For each successive cohort of patients, we use adaptive randomization to choose dose–schedule combinations based on their estimated utility values, subject to minimum toxicity and efficacy requirements. At the end of the trial, a final recommendation is made for each subgroup. While this work focuses on biomarker‐based stratification, the proposed design is readily applicable to other patient subgroups without modification.

Several trial designs have been suggested for determining the optimal dose and schedule, with different approaches proposed by various researchers. For example, Braun et al. [30] proposed a phase I clinical trial design that simultaneously optimizes both dose and schedule in terms of the overall risk of toxicity. Zhang and Braun [31] extend traditional approaches by incorporating adaptive variations to dose–schedule assignments within patients as the trial progresses. Lee et al. [32] introduced a phase I/II trial design that adaptively and dynamically optimizes each patient's dose in each of two cycles of therapy based on outcomes in each cycle. Guo et al. [33] developed a phase I/II trial design to find the optimal dose–schedule combination based on both toxicity and efficacy. Lin et al. [34] described a two‐stage phase I/II trial design to optimize dose–schedule within ordered disease subgroups, while Lin et al. [35] proposed a Bayesian adaptive basket trial design to optimize dose–schedule regimes within baskets. However, none of these designs take into account the interrelated factors of toxicity, efficacy, and immune response in immunotherapy to effectively leverage all available data information. As far as we know, the present article introduces the first phase I/II dose–schedule finding design for immunotherapy trials that seeks to determine the subgroup‐specific optimal dose–schedule combination by jointly accounting for immune response, toxicity, and efficacy.

The remainder of this article is organized as follows. In Section 2, we describe the probability models and present the dose–schedule finding algorithm. In Section 3, we investigate the operating characteristics of the proposed design through simulation studies. We provide concluding remarks in Section 4.

## 2 Method

### 2.1 Probability Models

Consider a phase I/II dose–schedule finding trial with J doses of an immunotherapeutic agent, $d_{1}<\cdots <d_{J}$, and K schedules, $s_{1},\ldots ,s_{K}$. Let dose–schedule combination (j,k) represent dose $d_{j}$ administered under schedule $s_{k}$, and let Z, X, and Y denote the immune response, toxicity outcome, and efficacy, respectively. We consider a binary biomarker with a subgroup indicator M=0 or 1 corresponding to the marker‐negative or marker‐positive subgroup, respectively, with the marker‐positive subgroup indicating improved immune response and efficacy outcome. The objective of the trial is to identify the optimal combination of dose and schedule for each marker subgroup in terms of toxicity, immune response, and efficacy.

#### 2.1.1 Immune Response Model

The immune response Z is taken to be the increase in a log‐transformed immune activity (e.g., T‐cell resistance) from baseline to post‐treatment, which is generally a continuous outcome. We assume the observed immune response Z of a patient in subgroup M treated at dose–schedule combination (j,k) follows a normal distribution with mean $\mu_{Z}\left((j,k),M\right)$ and variance $\sigma_{Z}^{2}$, that is,

(1) $$Z|(j,k),M∼N\left[\mu_{Z}\left((j,k),M\right),\sigma_{Z}^{2}\right].$$

Since the expected value $\mu_{Z}\left((j,k),M\right)$ represents the mean pre‐post difference in the immune activity, it is expected to be 0 when there is no drug, that is, d=0. To satisfy this constraint, we use the following plateau model for $\mu_{Z}\left((j,k),M\right)$

(2) $$\mu_{Z}\left((j,k),M\right)=\alpha_{k}\exp(\delta M)(1-\exp(-\nu d_{j})),$$

where $\alpha_{k}$ and $\alpha_{k}\exp(\delta )$ are the maximum mean immune responses that the immunotherapeutic agent possibly achieves for marker‐negative and positive subgroups, respectively, for schedule k. We restrict $\alpha_{k}>0$ and δ>0 to reflect that immunotherapy typically increases immune activity, so the mean increase is positive, and that marker‐positive patients tend to have a higher maximum mean immune response than marker‐negative patients. We constrain ν>0 such that the immune response increases or first increases and then plateaus when the dose increases for a given schedule in a subgroup. It can be easily seen that $\mu_{Z}\left((j,k),M\right)=0$ when $d_{j}=0$ in (2). The mean immune response model (2) provides a parsimonious yet flexible framework for modeling the dose–schedule immune response relationships for the two subgroups.

#### 2.1.2 Toxicity Model

We take the conventional setting of modeling toxicity as a binary outcome X, indicating the occurrence or absence of a dose‐limiting toxicity (DLT). As for most immunotherapeutic agents, the toxicity distribution of the agent in our motivating trial is believed to be homogeneous across subgroups. Let $p_{\left(j,k\right)}$ be the toxicity probability of dose–schedule combination (j,k). We model $p_{\left(j,k\right)}$ using the logistic model,

(3) $$\text{logit}\left\{p_{\left(j,k\right)}\right\}=\beta_{0,k}+\beta_{1}d_{j},$$

where $\beta_{0,k}$ is the intercept for schedule k, and $\beta_{1}$ is the dose effect. This parsimonious yet flexible parameterization can accommodate both complete ordering, where toxicity increases monotonically with dose and schedule, and partial ordering, where toxicity increases with dose within schedules but may not be strictly monotonic across schedules [36, 37, 38].

#### 2.1.3 Efficacy Model

The efficacy outcome Y is modeled as an ordinal outcome with Y=0,1,…,R corresponding to increasingly desirable efficacy. For example, Y=0,1, and 2 indicate progressive disease (PD), stable disease (SD), and complete remission or partial remission (CR/PR), respectively.

Let $q_{l,(j,k),M}$ be the probability of Y=l at dose–schedule combination (j,k) for biomarker subgroup M, that is, $q_{l,(j,k),M}=\mathrm{Pr}\left(Y=l|(j,k),M\right)$ for l=0,1,2, and let $\zeta_{(j,k),M}=\text{Logit}\left(q_{2,(j,k),M}\right)$ and $\xi_{(j,k),M}=\text{Logit}(q_{1,(j,k),M}+q_{2,(j,k),M})$ be logit transformations of $q_{2,(j,k),M}$ and $q_{1,(j,k),M}+q_{2,(j,k),M}$, respectively. Compared with the immune response and toxicity, the efficacy outcome is more complex and therefore requires a more advanced modeling approach. We model $\zeta_{(j,k),0}$, for the marker‐negative subgroup, using a Bayesian dynamic model as follows:

(4) $$\begin{array}{ll} \zeta_{(j,k),0}|\zeta_{(j-1,k),0} & ∼N\left[\zeta_{(j-1,k),0}+\gamma \left\{\mu_{Z}\left((j,k),0\right)\right.\right.\\ & \;\left.\left.-\mu_{Z}\left((j-1,k),0\right)\right\},\sigma_{E,0}^{2}\right]\\ & =N\left[\zeta_{(j-1,k),0}+\gamma \left\{\alpha_{k}\left(\exp(-\nu d_{j-1})\right.\right.\right.\\ & \;\left.\left.\left.-\exp(-\nu d_{j})\right)\right\},\sigma_{E,0}^{2}\right]\\ \zeta_{(1,k),0} & ∼N(\zeta_{(0,k),0},\tau^{2}), \end{array}$$

for j=2,…,J and k=1,…,K, where γ>0 is the average effect of the immune response, $\zeta_{(0,k),0}$ and $\tau^{2}$ are hyperparameters. This Bayesian dynamic model allows strength borrowing across dose–schedule combinations through centering the parameter associated with a dose–schedule combination ($\zeta_{(j,k),0}$) on that of the next lower dose in the same schedule ($\zeta_{(j-1,k),0}$), as well as the average immune response effect γ. The variance $\sigma_{E,0}^{2}$, or the resulting nonconstant $\zeta_{(j,k),0}-\zeta_{(j-1,k),0}-\gamma \left\{\mu_{Z}((j,k),0)-\mu_{Z}\left((j-1,k),0\right)\right\}$ across schedules, characterizes the interaction effect between the immune response and schedule. Hyperparameters $\zeta_{(0,k),0}$ and $\tau^{2}$ can be determined by consulting with clinicians. As $\zeta_{(0,k),0}$ is the prior mean of $\zeta_{(1,k),0}$, which is the logit of the probability of PR/CR at the lowest dose of schedule k for marker‐negative group, we can elicit $\zeta_{(0,k),0}$ based on the clinician's best guess of that probability, and $\tau^{2}$ reflects the uncertainty of this prior guess.

To model $\zeta_{(j,k),1}$, for the marker‐positive subgroup, we borrow information across subgroups as follows,

(5) $$\zeta_{(j,k),1}|\zeta_{(j,k),0}∼N(\zeta_{(j,k),0},\sigma_{E,1}^{2})I(\zeta_{(j,k),1}>\zeta_{(j,k),0}),$$

for j=2,…,J and k=1,…,K, where N(.)I(.) represents a truncated normal distribution with support specified in the indicator function. Model (5) ensures that the CR/PR probability is higher for the marker‐positive subgroup than the marker‐negative subgroup for any (j,k).

To model $\xi_{(j,k),M}$, note that $\zeta_{(j,k),M}=\text{Logit}\left(q_{2,(j,k),M}\right)=\text{Logit}\left(\mathrm{Pr}(Y=2|(j,k),M)\right)$ and $\xi_{(j,k),M}=\text{Logit}(q_{1,(j,k),M}+q_{2,(j,k),M})=\text{Logit}\left(\mathrm{Pr}(Y>0|(j,k),M)\right)$. Following the spirit of the proportional odds model, we assume

(6) $$\xi_{(j,k),M}=\lambda +\zeta_{(j,k),M},$$

where λ>0. Model (6), combined with (5), also ensures that the probability of SD/CR/PR is higher in the marker‐positive subgroup than in the marker‐negative subgroup for each dose–schedule combination (j,k).

According to our motivating trial, the treatment effect of the immunotherapy is believed to be mostly mediated by the immune response. Therefore, in the above model, we assume that conditional on the mean immune response, efficacy probability is independent of dose. For cases where such an assumption may not be true, we can add a dose in the model for $\zeta_{(j,k),0}$ as follows:

$$\begin{array}{l} \begin{array}{cc} \zeta_{(j,k),0}|\zeta_{(j-1,k),0} & ∼N\left[\zeta_{(j-1,k),0}+\gamma_{1}\left\{\mu_{Z}\left((j,k),0\right)\right.\right.\\ & \;\left.\left.-\mu_{Z}\left((j-1,k),0\right)\right\}+\gamma_{2}(d_{j}-d_{j-1}),\sigma_{E,0}^{2}\right]. \end{array} \end{array}$$

We do not consider the joint distribution of toxicity X and efficacy Y because previous research shows that such joint modeling does not improve the performance of the dose‐finding [39, 40], especially for immunotherapy agents whose efficacy tends to be weakly correlated with toxicity. Moreover, Cunanan and Koopmeiners [39] noted that, given the limited sample sizes typical of phase I/II trials, it is often challenging to accurately estimate the dependence or correlation between efficacy and toxicity in fully parametric joint models. Our approach of independent modeling simplifies the model estimation with little impact on the operating characteristics of the design, as will be shown by the simulation studies later.

#### 2.1.4 Likelihood

For the ith patient, denote the observed outcome by $D_{i}=(Z_{i},X_{i},Y_{i})$, the assigned dose–schedule combination by (j(i),k(i)), and the subgroup indicator by $M_{i}$. Let $\alpha =\{\alpha_{k}\}$, $\zeta =\{\zeta_{(j,k),M}\}$, $\beta_{0}=\{\beta_{0,k}\}$, and let $\Theta =(\delta ,\nu ,\sigma_{Z}^{2},\beta_{1},\gamma ,\lambda ,\alpha ,\zeta ,\beta_{0})$ represent all parameters. The likelihood for the ith patient is

$$L_{i}(D_{i};\Theta )=f(Z_{i}|\Theta )f(Y_{i}|\Theta )f(X_{i}|\Theta ),$$

where

$$f(Z_{i}|\Theta )=\phi \left(Z_{i};\alpha_{k(i)}\exp(\delta M_{i})(1-\exp(-\nu d_{j(i)})),\sigma_{Z}^{2}\right),$$

$$\begin{array}{ll} f(Y_{i}|\Theta ) & =I(Y_{i}=0)\left\{1-\text{expit}(\lambda +\zeta_{(j(i),k(i)),M_{i}})\right\}\\ & \;+I(Y_{i}=1)\left\{\text{expit}(\lambda +\zeta_{(j(i),k(i)),M_{i}})-\text{expit}(\zeta_{(j(i),k(i)),M_{i}})\right\}\\ & \;+I(Y_{i}=2)\text{expit}(\zeta_{(j(i),k(i)),M_{i}}), \end{array}$$

$$f(X_{i}|\Theta )=\frac{\exp\left\{X_{i}(\beta_{0,k(i)}+\beta_{1}d_{j(i)})\right\}}{1+\exp(\beta_{0,k(i)}+\beta_{1}d_{j(i)})},$$

$\phi (;\mu ,\sigma^{2})$ denotes the probability density function for a normal distribution with mean μ and variance $\sigma^{2}$, and expit(.)=exp(.)/(1+exp(.)) denotes the inverse of the logit transformation. Let n=1,…,N denote an interim sample size when a dose–schedule assignment decision is to be made during the trial, and ${𝒟}_{n}=(D_{1},\ldots ,D_{n})$ denote the observed data from the first n patients. The likelihood for the first n patients in the trial is $L({𝒟}_{n};\Theta )=\prod_{i=1}^{n}L_{i}(D_{i};\Theta )$.

The joint posterior distribution based on the data from the first n patients is

$$\begin{array}{ll} p(\Theta |{𝒟}_{n}) & \propto L({𝒟}_{n};\Theta )\\ & \;\times \overset{K}{\underset{k=1}{\prod }}\left[\phi \left\{\zeta_{(1,k),0};\zeta_{(0,k),0},\tau^{2}\right\}\overset{J}{\underset{j=2}{\prod }}\phi \left\{\zeta_{(j,k),0};\zeta_{(j-1,k),0}\right.\right.\\ & \;\left.\left.+\gamma \left\{\mu_{Z}\left((j,k),0\right)-\mu_{Z}\left((j-1,k),0\right)\right\},\sigma_{E,0}^{2}\right\}\right]\\ & \;\times \overset{K}{\underset{k=1}{\prod }}\overset{J}{\underset{j=1}{\prod }}\left[\phi \left\{\zeta_{(j,k),1};\zeta_{(j,k),0},\sigma_{E,1}^{2}\right\}I(\zeta_{(j,k),1}>\zeta_{(j,k),0})\right]\\ & \;p(\alpha )p(\delta )p(\nu )p(\sigma_{Z}^{2})p(\gamma )p(\lambda )p(\beta_{0})p(\beta_{1}), \end{array}$$

where p(.) denotes the prior distribution of a parameter. We sample from this posterior distribution using the Markov chain Monte Carlo algorithm with the Gibbs sampler.

### 2.2 Prior Distributions

Since $\alpha_{k}$ has the interpretation of the maximum mean immune response for marker‐negative patients for schedule k, and exp(δ) has the interpretation of the ratio of the maximum immune response of the marker‐positive patients relative to the marker‐negative patients, we elicit prior estimates of $\alpha_{k}$ and exp(δ) from clinicians, denoted as ${\hat{\alpha }}_{k}$ and $\hat{r}$, respectively. $\alpha_{k}$ and δ are assigned Gamma prior distributions with mean ${\hat{\alpha }}_{k}$ and $\log(\hat{r})$, respectively, and relatively large standard deviations (e.g., $3{\hat{\alpha }}_{k}$ and $3\log(\hat{r})$) to obtain vague priors. We assign $\sigma_{Z}^{2}$ a vague inverse Gamma prior distribution, for example, $\sigma_{Z}^{2}∼IG(0.1,0.1)$ so that data will dominate the posterior distribution.

To specify the prior distribution for ν, we use the idea of minimal prior knowledge described in Gelman et al. [41]. By rewriting model (2) as $\nu d_{j}=-\log\left(1-\frac{\mu_{Z}((j,k),M)}{\alpha_{k}\exp(\delta M)}\right)$, a change of 4.6 moves $\frac{\mu_{Z}((j,k),M)}{\alpha_{k}\exp(\delta M)}$ from 0.01 to 0.99. Considering the range of $\frac{\mu_{Z}((j,k),M)}{\alpha_{k}\exp(\delta M)}$ in (0,1), it is reasonable to assume that the effect of a covariate is unlikely to be more dramatic than that. Therefore, we scale $d_{j}$ to have standard deviation 0.5, and assign ν a truncated normal prior distribution $\nu ∼N(0,2.3^{2})I(\nu >0)$ so that a change in $d_{j}$ from one standard deviation below the mean to one standard deviation above the mean will most likely result in a difference of less than 4.6 for the regression in Equation (2).

In the toxicity model (3), we assign $\beta_{1}$ the Gelman prior Cauchy (0, 2.5) after scaling dose $d_{j}$ so that the standard deviation is 0.5 [41]. We let $\beta_{0,k}$ independently follow normal prior distributions N(−4,1) so that a priori the toxicity probability for each schedule is centered at 0.018 with 95% credible interval (0.0025,0.12) when there is no dose, that is, when $d_{j}=0$.

In the efficacy models, the prior specification of γ follows the spirit of Gelman et al. [41] for the logistic regression. Let $M_{\mu }$ be the maximum possible value of the mean immune response, $\mu_{Z}\left((j,k),M\right)$, across all dose–schedule combinations, elicited from clinicians. We assign γ a Cauchy(0,2.5) prior distribution after dividing each $\mu_{Z}\left((j,k),M\right)$ by $\frac{1}{2}M_{\mu }$ so that a change of $\mu_{Z}\left((j,k),M\right)$ from 0 to $\frac{1}{2}M_{\mu }$ or from $\frac{1}{2}M_{\mu }$ to $M_{\mu }$, will most likely result in a difference of less than 5 on the logit scale. We assign a uniform prior λ∼U(0,8) so that a reasonable range of $\xi_{(j,k),M}$, or equivalently Pr(Y>0|(j,k),M) is covered. For example, when Pr(Y=2|(j,k),M)=c, where c takes any value from 0.03 to 0.99, the support for Pr(Y>0|(j,k),M) is (c,0.99) under this prior.

### 2.3 Subgroup‐Specific Optimal Dose–Schedule Combination

We measure the desirability of a dose–schedule combination using a utility function that accounts for the risk‐benefit tradeoff between toxicity and efficacy. For patients in biomarker subgroup M, the true utility for a given dose–schedule combination (j,k) is defined as

$$\begin{array}{ll} U_{\text{true}}\left((j,k),M\right) & =q_{2,(j,k),M}+w_{1}q_{1,(j,k),M}\\ & \;-w_{2}p_{\left(j,k\right)}-w_{3}p_{\left(j,k\right)}I(p_{\left(j,k\right)}>\phi_{T}), \end{array}$$

where $0<w_{1}<1$, $w_{2}>0$, and $w_{3}>0$ are weights, and $\phi_{T}$ denotes the clinician‐specified upper limit of the toxicity rate. $w_{1}$ is a desirability weight to convert SD to CR/PR. For example, if two SDs are considered equivalent to a CR/PR, then $w_{1}=0.5$. So the first two terms, $q_{2,(j,k),M}+w_{1}q_{1,(j,k),M}$, convert the ordinal efficacy to the CR/PR rate. $w_{2}$ can be interpreted as the number of units of CR/PR that patients are willing to trade for one unit of decrease in toxicity. We add the last term, $w_{3}p_{\left(j,k\right)}I(p_{\left(j,k\right)}>\phi_{T})$, to reflect the practical consideration that a higher penalty on toxicity is required if it exceeds $\phi_{T}$. A large value of $w_{3}$ implies a higher preference for choosing doses with toxicity probabilities lower than $\phi_{T}$ [42]. Values of $w_{1},w_{2}$, and $w_{3}$ can be elicited from patients and/or clinicians. For our motivating trial, $w_{1}$ was determined to be 0.5 so that 2 SDs are equivalent to 1 CR/PR, which was based on the clinician's judgment and patients' preferences. $w_{2}$ and $w_{3}$ were determined to be $w_{2}=0.3$ and $w_{3}=1.2$, which represent 30% penalty for toxicity less than $\phi_{T}$, and a 150% penalty for toxicities greater than $\phi_{T}$.

During the trial, given the interim data ${𝒟}_{n}$ collected from n patients, we evaluate the desirability of dose–schedule combination (j,k) for patients in biomarker subgroup M based on the estimated utility,

$$\begin{array}{ll} U_{n}\left((j,k),M\right) & ={\hat{q}}_{2,(j,k),M}+w_{1}{\hat{q}}_{1,(j,k),M}\\ & \;-w_{2}{\hat{p}}_{\left(j,k\right)}-w_{3}{\hat{p}}_{\left(j,k\right)}I({\hat{p}}_{\left(j,k\right)}>\phi_{T}), \end{array}$$

where ${\hat{q}}_{2,(j,k),M},{\hat{q}}_{1,(j,k),M}$, and ${\hat{p}}_{\left(j,k\right)}$ are posterior means of the corresponding probabilities.

Let $\pi_{(j,k),M}=\mathrm{Pr}(Y>0|(j,k),M)$ denote the response rate of SD/PR/CR for subgroup M at dose–schedule combination (j,k), and let $\phi_{E}$ denote the lower limit of the response rate specified by physicians. We define the subgroup‐specific optimal dose schedule for subgroup M as the dose–schedule combination with the highest utility $U_{\text{true}}\left((j,k),M\right)$ while satisfying $p_{\left(j,k\right)}<\phi_{T}$ and $\pi_{(j,k),M}>\phi_{E}$. The definition of acceptable efficacy accounts for the practical consideration that, for an immunotherapeutic agent, a dose is considered promising if it can achieve a certain rate of CR/PR or a certain rate of SD (if the rate of CR/PR is low). SD is often regarded as a favorable outcome for immunotherapy, as many immunotherapeutic agents prolong survival without much tumor shrinkage. In practice, based on the observed data, posterior probabilities are used to construct admissible sets that satisfy the safety and efficacy requirements. Among these, the dose–schedule combination with the largest posterior mean utility is identified as the optimal dose–schedule combination. We illustrate the detailed design strategies and implementation procedures in the next section (Section 2.4).

### 2.4 Dose–Schedule Finding Algorithm

We propose a two‐stage design to determine the optimal dose–schedule combination for each subgroup. Stage I is based on only the toxicity outcome with the purpose of quickly exploring the dose–schedule space and accumulating preliminary data to facilitate stage II dose–schedule finding. In stage I, we independently escalate in each schedule until we reach a dose that violates the safety requirement $\mathrm{Pr}(p_{\left(j,k\right)}<\phi_{T}|{𝒟}_{n})>C_{I}$, where $C_{I}$ is a probability cutoff that will be tuned through simulation studies. More precisely,

1. For each schedule k,k=1,…,K, treat one cohort of patients at (1,k), that is, the lowest dose of schedule k.
2. For schedule k,k=1,…,K, if the highest tried dose $\left(h_{k},k\right)$ is unacceptably toxic or the highest dose J is reached, then schedule k is closed for stage I. If not, escalate to $\left(h_{k}+1,k\right)$ by assigning the next cohort of patients to combination $\left(h_{k}+1,k\right)$. If all schedules are closed, stage I is completed.

In stage I of the trial, data are sparse, making the estimates of the toxicity probabilities highly unreliable. As a result, we evaluate the safety requirement based on the simple Beta‐Binomial model in stage I. Precisely, we assume that the toxicity probability $p_{\left(j,k\right)}$ at dose–schedule combination (j,k) follows a Beta prior distribution $\text{Beta}(\kappa_{1},\kappa_{2})$, and the number of patients who experience toxicity $m_{\left(j,k\right)}$ among $n_{\left(j,k\right)}$ treated patients follows a binomial distribution $\text{Binom}(n_{\left(j,k\right)},p_{\left(j,k\right)})$. We set $\kappa_{1}=0.1$ and $\kappa_{2}=0.2$ so that the observed data dominate the posterior distribution. Under the Beta‐Binomial model, the posterior distribution of $p_{j,k}$ is $\text{Beta}(\kappa_{1}+m_{\left(j,k\right)},\kappa_{2}+n_{\left(j,k\right)}-m_{\left(j,k\right)})$. Since we assume the toxicity distribution is homogeneous across subgroups, subgroups are not considered in stage I. Although stage I does not utilize patient immune response or efficacy data, these data are collected to facilitate the model fitting in stage II.

In stage II, we adaptively randomize incoming patients to admissible dose–schedule combinations that satisfy the safety and efficacy requirements. Based on interim data ${𝒟}_{n}$, we define a dose–schedule combination (j,k) as admissible for biomarker subgroup M if it satisfies both the safety requirement

(7) $$\mathrm{Pr}(p_{\left(j,k\right)}<\phi_{T}|M,{𝒟}_{n})>C_{T},$$

and the efficacy requirement

(8) $$\mathrm{Pr}(\pi_{(j,k),M}>\phi_{E}|M,{𝒟}_{n})>C_{E},,$$

where $C_{T}$ and $C_{E}$ are prespecified toxicity and efficacy cutoffs, which should be calibrated to obtain good design operating characteristics. We denote the set of admissible dose–schedule combinations by ${𝒜}_{M,n}$ for subgroup M.

We treat patients in cohorts of size c. The dose–schedule finding design can be summarized as follows:

1. Based on the currently observed data ${𝒟}_{n}$, determine the admissible set ${𝒜}_{M,n}$ for each subgroup based on safety and efficacy requirements (7) and (8).
2. If ${𝒜}_{M,n}$ is empty for both subgroups, terminate the trial and conclude that no dose is acceptable for both subgroups. If ${𝒜}_{M,n}$ is empty for one of the subgroups, then conclude no dose is acceptable for that subgroup, and future patients in that subgroup are treated off protocol.
3. If ${𝒜}_{M,n}$ is not empty for at least one subgroup, then the patients in the next cohort in subgroup M with nonempty ${𝒜}_{M,n}$ are adaptively randomized to admissible dose–schedule combination $\left\{(j,k),(j,k)\in {𝒜}_{M,n}\right\}$ with randomization probability $\psi_{(j,k),M}$ proportional to its posterior mean utility, that is,
$$\psi_{(j,k),M}=\frac{U_{n}((j,k),M)}{\sum_{(j^{\prime },k^{\prime })\in {𝒜}_{M,n}}U_{n}((j^{\prime },k^{\prime }),M)}.$$
4. Repeat the above steps until reaching the maximum sample size N or terminating the trial early. Select the admissible dose–schedule combination with the largest posterior mean utility $U_{N}((j,k),M)$ as the subgroup‐specific optimal dose for subgroup M.

## 3 Simulation Study

We conducted extensive simulation studies to evaluate the performance of the proposed design. We considered a trial with three doses (0.1, 0.5, and 0.9) and three schedules, with a maximum sample size of 120 and 2 subgroups, treated in cohorts of size three. We also specified the upper limit for toxicity $\phi_{T}=0.3$, the lower limit for efficacy $\phi_{E}=0.3$, and the marker‐positive prevalence ϕ=0.5.

For each schedule k, we set ${\hat{\alpha }}_{k}=20$ as an elicited prior estimate of the maximum immune response for marker‐negative patients, and assign a vague prior $\alpha_{k}∼\text{Gamma}(1/9,1/180)$, so that the prior mean was 20 and the prior standard deviation was 60, which was 3 times the prior mean. The prior for δ was taken to be δ∼Gamma(1/9,5/18) to match the clinician‐elicited ratio of the maximum immune response of the marker‐positive patients relative to the marker‐negative patients of 1.5 ($\hat{r}=1.5$). Based on the clinician's prior knowledge, we set $\zeta_{(0,1),0}=\zeta_{(0,2),0}=\zeta_{(0,3),0}=-2.2$, and $\tau^{2}=1$, such that at the lowest dose level of each schedule, the prior probability of PR/CR was 0.1, and 2 standard deviations of the mean cover PR/CR probability from 0.01 to 0.45 for marker‐negative patients. The clinician‐elicited maximum possible mean immune response value $M_{\mu }=30$. We set $w_{1}=0.5$, $w_{2}=0.3$ and $w_{3}=1.2$. Probability cutoffs $C_{I}=0.05,C_{T}=0.2$, $C_{E}=0.2$, and hyperparameters $\sigma_{E,0}^{2}=0.3$ and $\sigma_{E,1}^{2}=0.3$ were tuned through simulations to obtain desirable operating characteristics of the design.

**FIGURE 1:**
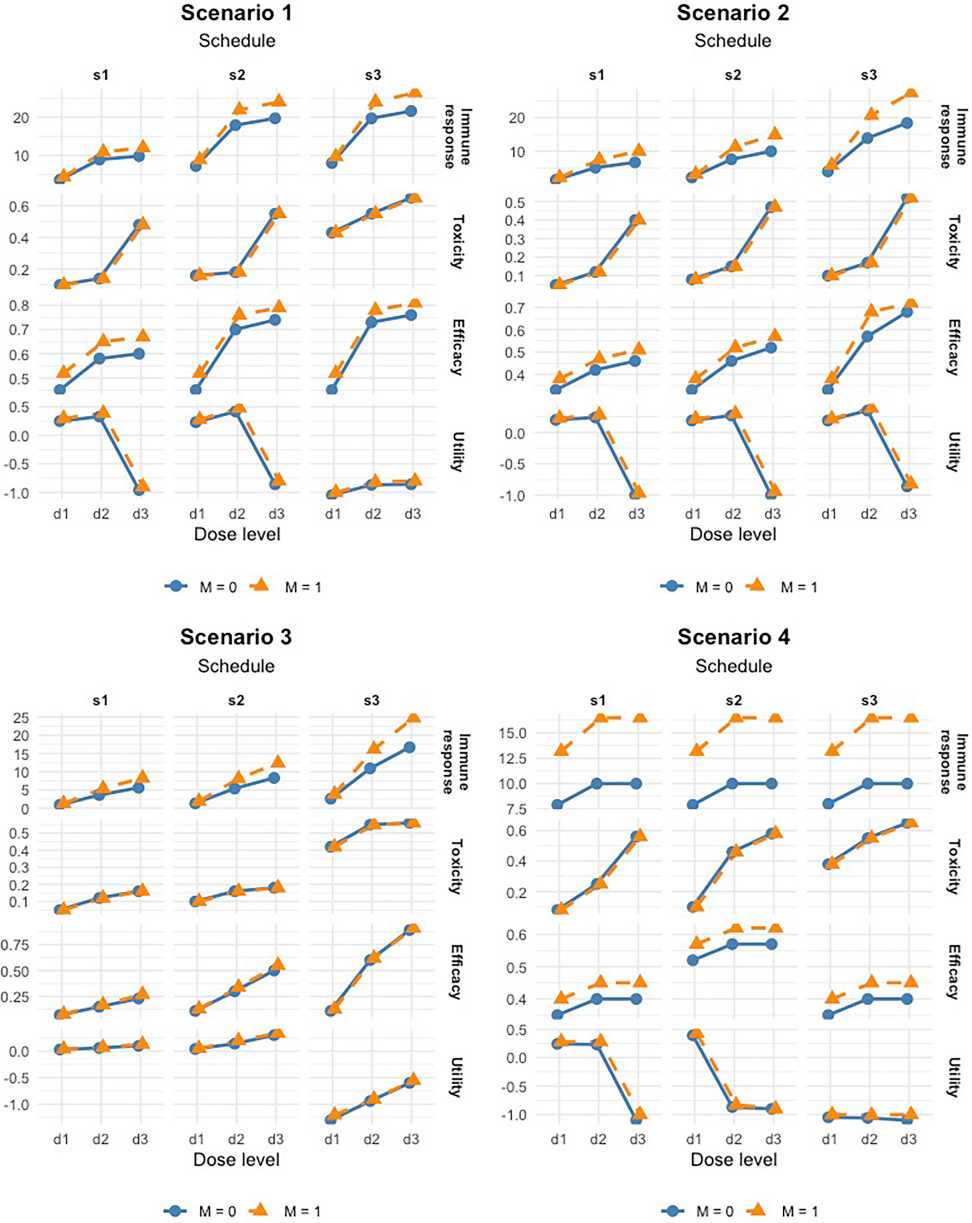
Dose‐response curves for the Scenarios 1–4 in Table 1.

**FIGURE 2:**
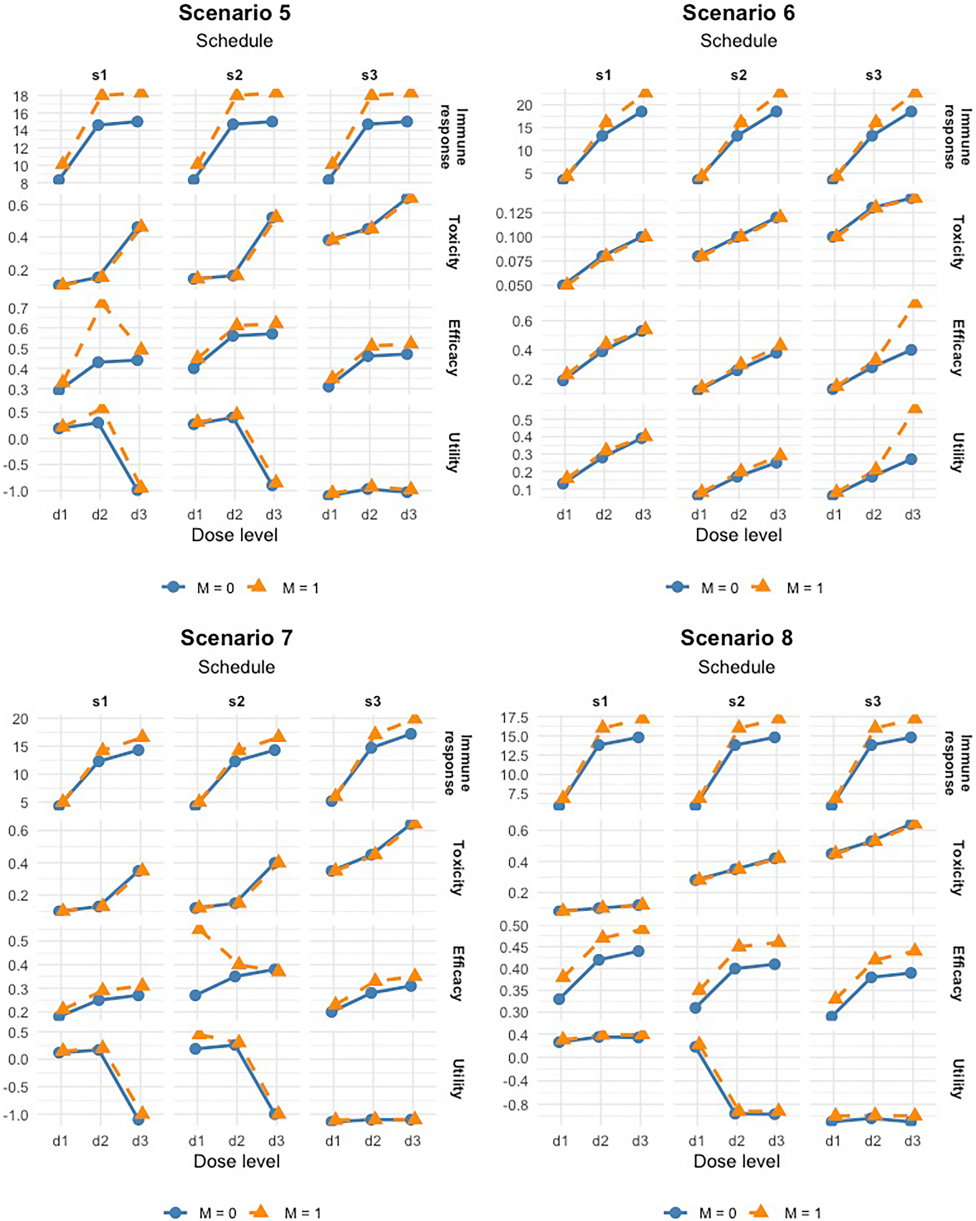
Dose‐response curves for the Scenarios 5–8 in Table 1.

We considered eight scenarios in our simulation studies, as shown in Table 1 and Table S1. Figures 1 and 2 show the true dose‐response curves for the mean immune response $\mu_{Z}\left((j,k),M\right)$, toxicity $p_{\left(j,k\right)}$, and efficacy $\pi_{(j,k),M}$ for each schedule and the two subgroups under these scenarios. The efficacy probabilities $q_{l,(j,k),M}$ were specified under the premise that when the immune response plateaus, efficacy plateaus or decreases, which is generally true for immunotherapy. Given the mean immune responses, the immune response $Z_{i}$ for patient i treated at dose–schedule combination (j(i),k(i)) in subgroup $M_{i}$ was generated from the normal model (1). Given the marginal toxicity and efficacy probabilities, the efficacy and toxicity data were generated from the Gumbel copula

$$C(u,v)=\exp\left[-{\left\{{\left(-log(u)\right)}^{\theta }+{\left(-log(v)\right)}^{\theta }\right\}}^{1/\theta }\right]$$

with correlation parameter θ=1.5 [43]. In Scenarios 1–4, both marker‐positive and marker‐negative subgroups share similar optimal dose–schedule combinations. In Scenarios 5 and 6, the subgroups have the same optimal dose level but differ in schedule. Scenario 7 features different optimal dose levels but a common schedule across subgroups, while Scenario 8 involves multiple optimal dose–schedule combinations for each subgroup.

**TABLE 1:** True immune response mean, toxicity and efficacy probabilities and utilities $\left(\mu_{Z},p,\pi ,U_{\text{true}}\right)$ under eight simulation scenarios, for each dose, schedule, and subgroup.

	M=0			M=1		
	$s_{1}$	$s_{2}$	$s_{3}$	$s_{1}$	$s_{2}$	$s_{3}$
Scenario 1						
$d_{1}$	(3.6, 0.10, 0.45, 0.25)	(7.2, 0.16, 0.45, 0.23)	(8.0, 0.43, 0.45, −1.05)	(4.4, 0.10, 0.52, 0.3)	(8.9, 0.16, 0.52, 0.28)	(9.7, 0.43, 0.52, −1.0)
$d_{2}$	(8.9, 0.14, 0.58, 0.33)	**(17.9, 0.18, 0.7, 0.42)**	(19.7, 0.55, 0.73, −0.87)	(10.9, 0.14, 0.65, 0.39)	**(21.9, 0.18, 0.76, 0.48)**	(24.0, 0.55, 0.78, −0.82)
$d_{3}$	(9.8, 0.48, 0.6, −0.96)	(19.7, 0.55, 0.74, −0.86)	(21.6, 0.65, 0.76, −0.86)	(12.0, 0.48, 0.67, −0.9)	(24.0, 0.55, 0.79, −0.8)	(26.4, 0.65, 0.81, −0.8)
Scenario 2						
$d_{1}$	(1.5, 0.05, 0.33, 0.2)	(2.2, 0.08, 0.33, 0.19)	(4.0, 0.1, 0.33, 0.19)	(2.2, 0.05, 0.38, 0.23)	(3.2, 0.08, 0.38, 0.22)	(5.9, 0.1, 0.38, 0.22)
$d_{2}$	(5.1, 0.12, 0.42, 0.24)	(7.6, 0.15, 0.46, 0.27)	**(13.9, 0.17, 0.57, 0.35)**	(7.5, 0.12, 0.47, 0.28)	(11.3, 0.15, 0.52, 0.3)	**(20.7, 0.17, 0.68, 0.39)**
$d_{3}$	(6.7, 0.4, 0.46, −1.0)	(10.0, 0.47, 0.52, −1.0)	(18.4, 0.52, 0.68, −0.86)	(10.0, 0.4, 0.51, −0.97)	(14.9, 0.47, 0.57, −0.94)	(27.4, 0.52, 0.72, −0.82)
Scenario 3						
$d_{1}$	(0.9, 0.05, 0.07, 0.03)	(1.3, 0.1, 0.11, 0.04)	(2.6, 0.42, 0.11, −1.3)	(1.3, 0.05, 0.08, 0.04)	(1.9, 0.1, 0.13, 0.05)	(3.9, 0.42, 0.13, −1.2)
$d_{2}$	(3.6, 0.12, 0.15, 0.06)	(5.4, 0.16, 0.3, 0.14)	(10.9, 0.55, 0.6, −0.94)	(5.4, 0.12, 0.17, 0.07)	(8.1, 0.16, 0.34, 0.2)	(16.2, 0.55, 0.62, −0.9)
$d_{3}$	(5.6, 0.16, 0.23, 0.1)	**(8.3, 0.18, 0.5, 0.3)**	(16.7, 0.56, 0.89, −0.6)	(8.3, 0.16, 0.27, 0.13)	**(12.4, 0.18, 0.55, 0.34)**	(24.8, 0.56, 0.91, −0.55)
Scenario 4						
$d_{1}$	(7.9, 0.08, 0.35, 0.24)	**(7.9, 0.1, 0.52, 0.39)**	(8.0, 0.38, 0.35, −1.05)	(13.2, 0.08, 0.4, 0.28)	**(13.2, 0.1, 0.57, 0.43)**	(13.2, 0.38, 0.4, −1.0)
$d_{2}$	(10.0, 0.25, 0.4, 0.23)	(10.0, 0.46, 0.57, −0.87)	(10.0, 0.55, 0.4, −1.06)	(16.5, 0.25, 0.45, 0.28)	(16.5, 0.46, 0.62, −0.83)	(16.5, 0.55, 0.45, −1.01)
$d_{3}$	(10.0, 0.56, 0.4, −1.1)	(10.0, 0.58, 0.57, −0.9)	(10.0, 0.65, 0.4, −1.1)	(16.5, 0.56, 0.45, −1.0)	(16.5, 0.58, 0.62, −0.9)	(16.5, 0.65, 0.45, −1.0)
Scenario 5						
$d_{1}$	(8.3, 0.1, 0.29, 0.19)	(8.3, 0.14, 0.4, 0.27)	(8.3, 0.38, 0.31, −1.1)	(10.1, 0.1, 0.33, 0.22)	(10.1, 0.14, 0.45, 0.31)	(10.1, 0.38, 0.35, −1.05)
$d_{2}$	(14.6, 0.15, 0.43, 0.3)	**(14.7, 0.16, 0.56, 0.4)**	(14.7, 0.45, 0.46, −0.97)	**(18.0, 0.15, 0.72, 0.57)**	(18.0, 0.16, 0.61, 0.45)	(18.0, 0.45, 0.51, −0.93)
$d_{3}$	(15.0, 0.46, 0.44, −0.99)	(15.0, 0.52, 0.57, −0.9)	(15.0, 0.64, 0.47, −1.03)	(18.3, 0.46, 0.49, −0.95)	(18.3, 0.52, 0.62, −0.85)	(18.3, 0.64, 0.52, −0.98)
Scenario 6						
$d_{1}$	(3.5, 0.05, 0.19, 0.13)	(3.5, 0.08, 0.12, 0.06)	(3.5, 0.1, 0.13, 0.06)	(4.3, 0.05, 0.23, 0.16)	(4.3, 0.08, 0.14, 0.08)	(4.3, 0.1, 0.15, 0.08)
$d_{2}$	(13.2, 0.08, 0.39, 0.28)	(13.2, 0.1, 0.26, 0.17)	(13.2, 0.13, 0.28, 0.17)	(16.1, 0.08, 0.44, 0.32)	(16.1, 0.1, 0.3, 0.2)	(16.1, 0.13, 0.33, 0.21)
$d_{3}$	**(18.5, 0.1, 0.53, 0.39)**	(18.5, 0.12, 0.38, 0.25)	(18.5, 0.14, 0.4, 0.27)	(22.6, 0.1, 0.54, 0.4)	(22.6, 0.12, 0.43, 0.29)	**(22.6, 0.14, 0.72, 0.56)**
Scenario 7						
$d_{1}$	(4.3, 0.1, 0.18, 0.12)	(4.3, 0.12, 0.27, 0.19)	(5.2, 0.35, 0.2, −1.14)	(5.0, 0.1, 0.21, 0.15)	**(5.0, 0.12, 0.55, 0.45)**	(6.0, 0.35, 0.23, −1.11)
$d_{2}$	(12.3, 0.13, 0.25, 0.17)	**(12.3, 0.15, 0.35, 0.26)**	(14.7, 0.45, 0.28, −1.1)	(14.2, 0.13, 0.29, 0.2)	(14.2, 0.15, 0.4, 0.3)	(17.1, 0.45, 0.33, −1.1)
$d_{3}$	(14.3, 0.35, 0.27, −1.1)	(14.3, 0.4, 0.38, −1.0)	(17.2, 0.64, 0.31, −1.1)	(16.6, 0.35, 0.31, −1.0)	(16.6, 0.4, 0.37, −1.0)	(19.9, 0.64, 0.35, −1.1)
Scenario 8						
$d_{1}$	(5.9, 0.08, 0.33, 0.26)	(5.9, 0.28, 0.31, 0.18)	(5.9, 0.45, 0.29, −1.1)	(6.9, 0.08, 0.38, 0.3)	(6.9, 0.28, 0.35, 0.22)	(6.9, 0.45, 0.33, −1.0)
$d_{2}$	**(13.8, 0.1, 0.42, 0.34)**	(13.8, 0.35, 0.4, −0.96)	(13.8, 0.53, 0.38, −1.04)	**(16.0, 0.1, 0.47, 0.38)**	(16.0, 0.35, 0.45, −0.92)	(16.0, 0.53, 0.42, −0.99)
$d_{3}$	**(14.8, 0.12, 0.44, 0.34)**	(14.8, 0.42, 0.41, −0.97)	(14.8, 0.64, 0.39, −1.1)	**(17.2, 0.12, 0.49, 0.39)**	(17.2, 0.42, 0.46, −0.92)	(17.2, 0.64, 0.44, −1.0)

*Note:* The values in boldface correspond to the subgroup‐specific optimal dose‐schedule combination.

We compared our proposed design with two alternative designs. The first alternative design (Alternative 1) ignored the immune response and considered only toxicity and efficacy, with the efficacy model (4) changed accordingly as

$$\begin{array}{ll} \zeta_{(j,k),0}|\zeta_{(j-1,k),0} & ∼N\left[\zeta_{(j-1,k),0}+\gamma \left\{d_{j}-d_{j-1})\right\},\sigma_{E,0}^{2}\right]\\ \zeta_{(1,k),0} & ∼N(\zeta_{(0,k),0},\tau^{2}), \end{array}$$

with the mean immune response replaced with the dose. The second alternative design (Alternative 2) used a parametric proportional odds model for efficacy.

$$\begin{array}{ll} \text{logit}\left[\mathrm{Pr}(Y\leq l|(j,k),M)\right] & =\gamma_{0,l,k,M}+\gamma_{1}\mu_{Z}\left((j,k),M\right)\\ & \;+\gamma_{2}\mu_{Z}{\left((j,k),M\right)}^{2},\;\;\;\;l=1,\ldots ,R-1 \end{array}$$

where $\gamma_{0,1,k,M}<\cdots <\gamma_{0,R-1,k,M}$ are intercepts for schedule k in subgroup M with order constraint $\gamma_{0,1,k,0}>\gamma_{0,1,k,1}$, and $\gamma_{1}$ is the dose effect.

Table 2 presents the selection percentages and the average number of patients treated at each dose–schedule combination across subgroups for all eight scenarios, comparing the proposed design with two alternative approaches. Values in boldface correspond to the true optimal dose–schedule combination. In general, the proposed design achieves superior performance in both correctly identifying the optimal dose–schedule combinations and allocating a greater number of patients to those combinations, followed by the Alternative 2 design, whereas the Alternative 1 design yields the least desirable performance. The superior performance of the proposed design is primarily due to its ability to incorporate immune response data and its use of a flexible dynamic modeling framework, which contrasts with the ignorance of immune response information in Alternative 1 and the more rigid parametric proportional odds model employed in Alternative 2.

**TABLE 2:** Simulation results: selection percentage and average number of patients treated at each dose–schedule combination.

Design		M=0			M=1		
		$s_{1}$	$s_{2}$	$s_{3}$	$s_{1}$	$s_{2}$	$s_{3}$
Scenario 1							
Proposed	$d_{1}$	6.1 (10.7)	6.0 (11.0)	0.6 (7.7)	7.0 (11.1)	6.2 (11.0)	0.7 (7.7)
	$d_{2}$	22.6 (11.5)	**61.9 (11.6)**	0.4 (2.1)	23.0 (11.3)	**60.1 (11.2)**	0.4 (2.1)
	$d_{3}$	1.7 (2.5)	0.7 (1.9)	0.0 (1.1)	2.1 (2.4)	0.5 (1.9)	0.0 (1.2)
Alternative 1	$d_{1}$	5.6 (8.6)	5.6 (9.3)	1.9 (6.4)	7.0 (8.6)	5.7 (9.3)	2.0 (6.7)
	$d_{2}$	18.4 (8.4)	**43.3 (8.5)**	0.3 (1.7)	17.6 (8.7)	**43.0 (8.4)**	0.3 (1.6)
	$d_{3}$	3.1 (2.0)	1.1 (1.5)	0.0 (1.0)	2.6 (2.0)	1.1 (1.5)	0.0 (0.9)
Alternative 2	$d_{1}$	10.4 (11.4)	7.7 (10.9)	2.8 (7.1)	6.5 (11.5)	6.8 (11.1)	2.1 (8.3)
	$d_{2}$	25.5 (11.3)	**53.0 (11.9)**	0.1 (2.0)	26.6 (11.3)	**57.3 (10.6)**	0.1 (2.0)
	$d_{3}$	0.0 (2.2)	0.5 (1.8)	0.0 (1.2)	0.1 (2.4)	0.5 (1.8)	0.0 (1.2)
Scenario 2							
Proposed	$d_{1}$	3.8 (7.3)	4.2 (7.6)	2.7 (7.6)	4.8 (8.2)	4.6 (8.0)	2.6 (8.0)
	$d_{2}$	9.1 (8.7)	15.0 (9.4)	**60.9 (11.8)**	8.5 (8.8)	14.6 (9.1)	**60.2 (11.0)**
	$d_{3}$	1.6 (2.9)	1.3 (2.3)	1.4 (2.1)	2.0 (2.8)	1.3 (2.3)	1.4 (2.0)
Alternative 1	$d_{1}$	3.2 (5.0)	3.9 (5.6)	3.2 (6.7)	3.2 (5.6)	4.8 (5.9)	3.1 (6.6)
	$d_{2}$	7.5 (6.0)	13.1 (6.6)	**36.8 (7.9)**	7.5 (6.3)	11.7 (6.4)	**37.4 (7.4)**
	$d_{3}$	2.1 (2.0)	1.2 (1.6)	0.8 (1.5)	2.1 (2.0)	1.2 (1.7)	0.8 (1.4)
Alternative 2	$d_{1}$	7.9 (7.0)	7.5 (7.7)	5.9 (8.8)	4.3 (8.1)	4.2 (8.6)	4.8 (9.0)
	$d_{2}$	10.6 (7.6)	18.2 (9.1)	**47.7 (12.1)**	10.2 (8.4)	20.0 (9.3)	**54.4 (10.3)**
	$d_{3}$	0.3 (2.8)	0.2 (2.2)	0.9 (2.1)	0.6 (2.7)	0.3 (2.2)	1.2 (1.9)
Scenario 3							
Proposed	$d_{1}$	0.0 (1.7)	0.1 (2.3)	0.1 (3.5)	0.0 (2.4)	0.2 (4.5)	0.1 (5.5)
	$d_{2}$	0.1 (2.4)	13.8 (14.1)	0.8 (4.1)	0.8 (4.7)	18.8 (15.1)	0.3 (3.0)
	$d_{3}$	2.6 (4.8)	**66.1 (19.9)**	0.0 (1.3)	4.2 (6.1)	**63.8 (13.8**)	0.1 (1.3)
Alternative 1	$d_{1}$	0.1 (4.6)	0.1 (5.7)	0.1 (6.0)	0.0 (5.8)	0.1 (6.7)	0.1 (6.1)
	$d_{2}$	5.0 (9.4)	17.2 (11.6)	1.3 (2.8)	4.0 (9.2)	15.5 (10.7)	1.3 (2.7)
	$d_{3}$	22.0 (9.6)	**54.0 (9.4)**	0.2 (1.3)	23.6 (8.8)	**55.2 (8.4**)	0.2 (1.3)
Alternative 2	$d_{1}$	0.0 (1.6)	0.1 (2.5)	0.1 (3.4)	0.0 (2.5)	0.1 (4.6)	1.1 (6.7)
	$d_{2}$	0.0 (1.8)	4.9 (8.9)	0.7 (5.1)	0.4 (5.0)	17.3 (15.1)	0.2 (2.8)
	$d_{3}$	0.5 (2.7)	**49.1 (15.9)**	0.0 (1.5)	6.0 (6.7)	**65.8 (14.2**)	0.0 (1.3)
Scenario 4							
Proposed	$d_{1}$	6.1 (11.2)	**81.0 (20.9)**	1.6 (8.4)	7.0 (12.4)	**79.6 (18.7)**	2.1 (9.2)
	$d_{2}$	5.1 (8.3)	6.2 (5.4)	0.0 (1.9)	5.8 (8.4)	5.5 (4.9)	0.0 (1.9)
	$d_{3}$	0.0 (1.6)	0.0 (1.4)	0.0 (1.2)	0.0 (1.6)	0.0 (1.4)	0.0 (1.2)
Alternative 1	$d_{1}$	6.1 (10.8)	**76.8 (20.7)**	5.0 (8.6)	6.3 (12.1)	**76.4 (18.8)**	5.2 (9.5)
	$d_{2}$	6.0 (8.1)	6.1 (5.5)	0.0 (2.0)	6.5 (8.3)	5.6 (5.2)	0.0 (1.9)
	$d_{3}$	0.0 (1.6)	0.0 (1.4)	0.0 (1.2)	0.0 (1.6)	0.0 (1.4)	0.0 (1.2)
Alternative 2	$d_{1}$	12.3 (11.5)	**81.5 (21.8)**	4.4 (9.1)	10.6 (12.9)	**81.2 (18.6)**	3.6 (10.2)
	$d_{2}$	0.1 (6.2)	0.1 (5.1)	0.0 (1.8)	1.8 (7.2)	1.8 (4.7)	1.0 (1.9)
	$d_{3}$	0.0 (1.6)	0.0 (1.4)	0.0 (1.2)	0.0 (1.6)	0.0 (1.4)	0.0 (1.2)
Scenario 5							
Proposed	$d_{1}$	1.0 (6.9)	9.1 (10.2)	0.7 (5.6)	0.7 (8.3)	7.2 (10.6)	0.5 (6.7)
	$d_{2}$	32.8 (14.2)	**54.1 (14.1)**	0.6 (2.9)	**53.4 (13.6)**	36.6 (12.4)	0.5 (2.9)
	$d_{3}$	0.8 (2.5)	0.9 (2.1)	0.0 (1.3)	0.3 (2.4)	0.8 (2.1)	0.0 (1.3)
Alternative 1	$d_{1}$	1.3 (8.4)	11.5 (11.7)	1.3 (6.3)	0.3 (9.1)	8.7 (11.6)	1.2 (7.2)
	$d_{2}$	32.4 (12.7)	**49.6 (12.2)**	0.2 (2.5)	**52.1 (12.7)**	34.6 (11.1)	0.1 (2.5)
	$d_{3}$	2.5 (2.6)	1.2 (2.1)	0.0 (1.3)	1.8 (2.5)	1.2 (2.0)	0.0 (1.3)
Alternative 2	$d_{1}$	1.7 (6.9)	13.0 (12.4)	2.3 (6.3)	1.3 (9.8)	10.3 (11.6)	0.8 (7.6)
	$d_{2}$	24.5 (11.5)	**56.0 (13.8)**	0.4 (2.5)	**32.2 (11.7)**	54.8 (11.3)	0.5 (2.6)
	$d_{3}$	0.0 (2.2)	0.0 (2.1)	0.0 (1.3)	0.0 (2.4)	0.1 (1.9)	0.0 (1.3)
Scenario 6							
Proposed	$d_{1}$	0.5 (3.4)	0.0 (2.0)	0.1 (2.3)	0.5 (4.6)	0.0 (2.7)	0.1 (3.5)
	$d_{2}$	4.9 (9.3)	0.4 (4.8)	0.4 (6.8)	6.0 (8.8)	0.8 (5.7)	0.7 (7.3)
	$d_{3}$	**53.9 (12.6)**	5.2 (7.7)	33.3 (10.8)	34.2 (10.1)	4.2 (7.2)	**52.8 (9.7)**
Alternative 1	$d_{1}$	0.5 (3.8)	0.0 (2.1)	0.0 (2.3)	0.5 (5.3)	0.1 (3.1)	0.0 (3.8)
	$d_{2}$	3.5 (9.0)	0.2 (4.2)	0.5 (6.2)	4.9 (8.4)	0.3 (5.4)	0.5 (6.6)
	$d_{3}$	**52.4 (12.1)**	8.7 (8.8)	33.5 (11.1)	37.6 (10.4)	5.0 (7.1)	**50.5 (9.9)**
Alternative 2	$d_{1}$	0.2 (3.7)	0.0 (1.9)	0.0 (2.3)	0.2 (4.8)	0.0 (2.8)	0.2 (3.9)
	$d_{2}$	2.0 (9.5)	0.2 (3.8)	0.3 (5.2)	3.5 (8.7)	0.6 (5.6)	1.1 (7.4)
	$d_{3}$	**62.8 (14.7)**	10.7 (7.4)	19.3 (9.2)	51.5 (10.7)	8.7 (7.4)	**34.2 (8.9)**
Scenario 7							
Proposed	$d_{1}$	0.6 (3.5)	37.5 (16.4)	0.9 (4.4)	1.0 (5.2)	**66.5 (17.1)**	0.5 (5.9)
	$d_{2}$	5.6 (6.2)	**43.5 (15.3)**	0.9 (3.0)	5.2 (7.6)	20.5 (13.1)	0.4 (2.8)
	$d_{3}$	2.2 (3.5)	3.2 (3.3)	0.0 (1.4)	1.8 (3.2)	1.3 (3.0)	0.0 (1.4)
Alternative 1	$d_{1}$	0.9 (3.7)	40.5 (16.7)	1.4 (4.8)	1.8 (5.4)	**68.6 (17.0)**	1.5 (6.3)
	$d_{2}$	4.2 (5.5)	**33.1 (14.9)**	1.0 (2.8)	4.1 (7.1)	17.7 (13.1)	0.4 (2.8)
	$d_{3}$	3.7 (3.5)	2.9 (3.2)	0.0 (1.4)	1.5 (3.2)	1.0 (3.0)	0.0 (1.4)
Alternative 2	$d_{1}$	3.2 (4.4)	23.9 (13.1)	4.7 (5.9)	3.3 (7.3)	**51.2 (14.3)**	2.7 (7.6)
	$d_{2}$	4.1 (5.2)	**32.8 (12.5)**	0.9 (2.8)	7.0 (8.0)	31.3 (12.4)	0.3 (2.8)
	$d_{3}$	0.9 (2.8)	1.2 (2.9)	0.0 (1.3)	1.2 (3.0)	2.1 (2.9)	0.0 (1.4)
Scenario 8							
Proposed	$d_{1}$	10.4 (8.6)	6.9 (7.4)	1.0 (4.7)	12.3 (10.1)	6.3 (8.3)	0.9 (5.6)
	$d_{2}$	**45.7 (16.5**)	5.8 (6.5)	0.1 (2.2)	**44.1 (14.8)**	5.7 (5.9)	0.1 (2.2)
	$d_{3}$	**28.9 (10.8)**	0.2 (1.9)	0.0 (1.2)	**29.7 (9.8)**	0.2 (1.8)	0.0 (1.2)
Alternative 1	$d_{1}$	11.0 (9.6)	6.2 (8.0)	1.7 (5.4)	13.9 (10.4)	7.7 (8.9)	1.6 (6.2)
	$d_{2}$	**33.9 (13.8**)	4.4 (5.9)	0.3 (2.2)	**36.6 (13.1)**	5.3 (5.9)	0.3 (2.2)
	$d_{3}$	**40.8 (11.6)**	0.3 (2.0)	0.0 (1.2)	**33.8 (9.9)**	0.4 (1.9)	0.0 (1.1)
Alternative 2	$d_{1}$	14.7 (9.5)	13.9 (9.6)	3.4 (5.7)	16.0 (10.6)	13.9 (9.7)	2.9 (6.8)
	$d_{2}$	**36.9 (12.7**)	7.1 (6.1)	0.0 (2.2)	**39.4 (13.1)**	7.0 (5.8)	0.0 (2.2)
	$d_{3}$	**16.0 (8.6)**	0.2 (1.8)	0.0 (1.2)	**20.6 (8.9)**	0.1 (1.9)	0.0 (1.2)

For example, in Scenario 1, the mean immune response increases up to dose level $d_{2}$ and then plateaus. Once the immune response reaches a plateau, the CR/PR and SD/CR/PR rates also plateau across all schedules and biomarker subgroups, while toxicity continues to rise with increasing dose. As a result, $\left(d_{2},s_{2}\right)$ represents the optimal dose–schedule combination for all subgroups and yields the highest true utility. The proposed design correctly selects this combination in 60.2% of simulated trials for marker‐positive patients, which is 17.1% and 2.8% higher than the corresponding selection rates of Alternatives 1 and 2, respectively. Furthermore, the proposed design allocates an average of 11.6 patients to the optimal dose–schedule combination, which exceeds the average allocations under Alternatives 1 and 2 by 2.8 and 0.6 patients, respectively. Similar patterns are observed in the marker‐negative subgroup.

In Scenario 6, both the immune response and SD/CR/PR rates increase monotonically with dose within each schedule. Accordingly, the optimal dose–schedule combinations are $\left(d_{3},s_{1}\right)$ for the marker‐negative subgroup and $\left(d_{3},s_{3}\right)$ for the marker‐positive subgroup. Although Alternative 2 achieves better performance in the marker‐negative subgroup, it is less efficient in the marker‐positive subgroup.

In Scenario 7, under schedule 2 ($s_{2}$), the immune response plateaus at dose level $d_{2}$. However, the SD/PR/CR efficacy responses diverge by subgroup: the response plateaus at $d_{2}$ for marker‐negative patients but decreases monotonically for marker‐positive patients. Consequently, the optimal dose–schedule combinations are $\left(d_{2},s_{2}\right)$ for marker‐negative patients and $\left(d_{1},s_{2}\right)$ for marker‐positive patients. The proposed design continues to provide the highest accuracy in dose selection and patient allocation across subgroups. Consistent findings are observed in the remaining representative scenarios.

We performed additional simulation studies by expanding the dose–schedule space to include 4×3, 5×3, 4×4, and 4×5 dose–schedule combinations. We also included simulation scenarios under extreme conditions where all doses exhibited excessive toxicity to assess the design's safety protection. The scenarios and results are summarized in Tables S6 and S7 and Figures S1 and S2. The proposed design continues to demonstrate robust and desirable performance in identifying optimal dose–schedule combinations and efficiently allocating patients to those regimens. In addition, the design can terminate trials efficiently with smaller sample sizes and make correct decisions under overly toxic scenarios.

### 3.1 Sensitivity Analysis

We performed comprehensive sensitivity analyses to assess the robustness of the proposed design with respect to the prevalence of marker‐positive patients, total sample size, and the specification of noninformative prior distributions.

The simulation results, summarized in Tables S2–S4, indicate that the proposed design is not sensitive to variations in marker‐positive prevalence probabilities. As expected, the operating characteristics improve with increasing sample size. To evaluate sensitivity to prior assumptions, we specified diffuse priors by inflating the standard deviations of $\alpha_{k}$ and δ to five times their clinician‐elicited estimates. Additional prior specifications included $\sigma_{Z}^{2}∼\text{IG}(0.01,0.01)$, $\nu ∼N(0,4.6^{2})\;I(\nu >0)$, γ∼Cauchy(0,5), λ∼Uniform(0,16), $\beta_{1}∼\text{Cauchy}(0,5)$, and $\beta_{0,k}∼N(-4,2)$. The results remained consistent with the original results from the Table 2, suggesting that the proposed design is robust to prior specification.

The Supporting Information available online also include extensive sensitivity analyses evaluating the performance of the proposed design under a nonparametric optimal benchmark comparison [44] and across randomly generated scenarios.

## 4 Discussion

We have proposed a Bayesian biomarker‐based phase I/II clinical trial design to determine the subgroup‐specific optimal dose–schedule combination for immunotherapy. We develop parametric models for the immune response and toxicity. Due to the complex dose‐efficacy relationships, rather than imposing stringent model assumptions on the dose‐efficacy curve, we utilize flexible Bayesian dynamic models for efficacy to borrow information across dose–schedule combinations. Simulation studies show that the proposed design has desirable operating characteristics and yields robust performance across many shapes of the underlying dose‐efficacy curves.

In this article, we assume there are two predetermined subgroups. In some situations, the subgroups may be unknown. In such cases, there is a significant interest in integrating longitudinal biomarker data or multiple biomarker measurements to facilitate the identification of subgroups. In addition, as in most current phase I/II trial designs, we assume the outcomes are quickly ascertainable. For some immunotherapy trials, the toxicity and/or efficacy may be late‐onset and require a longer time to score. This will cause accrual suspension as the outcomes may not be observed soon enough to apply decision rules to choose treatments for new patients. One approach to address this issue is to apply the methodology proposed by Liu et al. [45] or Jin et al. [46], which accommodates delayed outcomes using Bayesian data augmentation or the expectation‐maximization algorithm. These are areas of our future research. The total sample size in adaptive dose–schedule trials is typically determined through simulation rather than analytical calculation, as it depends on the complexity of the design and operational constraints such as accrual rate and the number of dose–schedule combinations under investigation. In practice, we recommend that investigators conduct simulation‐based calibration to identify a sample size that balances trial efficiency with statistical reliability. The software used to simulate and implement the proposed design is publicly available at: https://github.com/FrankQiu20/Biomarker‐Based‐Dose‐Schedule‐Optimization‐Design. A detailed, step‐by‐step tutorial and example codes are also provided in the Supporting Information to facilitate reproducibility and practical application.

## Funding

This work was supported by the National Cancer Institute (Grant No. P30CA142543) and the Indiana Clinical and Translational Sciences Institute (Grant Number UM1TR004402).

## Conflicts of Interest

The authors declare no conflicts of interest.

## Supporting information




**Data S1.** Supporting Information.

## Data Availability

Data sharing not applicable to this article as no datasets were generated or analyzed during the current study.

## References

[1] J. Galon , A. Costes , F. Sanchez‐Cabo , et al., “Type, Density, and Location of Immune Cells Within Human Colorectal Tumors Predict Clinical Outcome,” Science 29 (2006): 1960–1964. 10.1126/science.1129139 17008531

[2] S. Walter , T. Weinschenk , A. Stenzl , et al., “Multipeptide Immune Response to Cancer Vaccine IMA901 After Single‐Dose Cyclophosphamide Associates With Longer Patient Survival,” Nature Medicine 18 (2012): 1254–1261. 10.1038/nm.2883 22842478

[3] T. C. Zuiverloon , A. J. Nieuweboer , H. Vekony , W. J. Kirkels , C. H. Bangma , and E. C. Zwarthoff , “Markers Predicting Response to Bacillus Calmette‐Guerin Immunotherapy in High‐Risk Bladder Cancer Patients: A Systematic Review,” European Urology 61 (2012): 128–145. 22000498 10.1016/j.eururo.2011.09.026

[4] S. Liu , B. Guo , and Y. Yuan , “A Bayesian Phase I/II Trial Design for Immunotherapy,” Journal of the American Statistical Association 113 (2018): 1016–1027. 31741544 10.1080/01621459.2017.1383260 PMC6860919

[5] B. Guo , D. Li , and Y. Yuan , “SPIRIT: A Seamless Phase I/II Randomized Design for Immunotherapy Trials,” Pharmaceutical Statistics 17 (2018): 527–540. 29882388 10.1002/pst.1869

[6] C. Wang , G. Rosner , and R. Roden , “A Bayesian Design for Phase I Cancer Therapeutic Vaccine Trials,” Statistics in Medicine 38 (2019): 1170–1189. 30368868 10.1002/sim.8021 PMC6399043

[7] B. Guo , Y. Park , and S. Liu , “A Utility‐Based Bayesian Phase I‐II Design for Immunotherapy Trials With Progression‐Free Survival End Point,” Journal of the Royal Statistical Society: Series C: Applied Statistics 68 (2019): 411–425.

[8] B. Guo , E. Garrett‐Mayer , and S. Liu , “A Bayesian Phase I/II Design for Cancer Clinical Trials Combining an Immunotherapeutic Agent With a Chemotherapeutic Agent,” Journal of the Royal Statistical Society: Series C: Applied Statistics 70 (2021): 1210–1229.

[9] H. Shi , J. Cao , Y. Yuan , and R. Lin , “uTPI: A Utility‐Based Toxicity Probability Interval Design for Phase I/II Dose‐Finding Trials,” Statistics in Medicine 40 (2021): 2626–2649. 33650708 10.1002/sim.8922 PMC13138761

[10] S. Topalian , F. Hodi , J. Brahmer , et al., “Safety, Activity, and Immune Correlates of Anti‐PD‐1 Antibody in Cancer,” New England Journal of Medicine 366 (2012): 2443–2454. 22658127 10.1056/NEJMoa1200690 PMC3544539

[11] K. Mahoney and M. Atkins , “Prognostic and Predictive Markers for the New Immunotherapies,” Oncology 28 (2014): 39–48. 25384886

[12] E. Garon , N. Rizvi , R. Hui , et al., “Pembrolizumab for the Treatment of Non‐Small‐Cell Lung Cancer,” New England Journal of Medicine 372 (2015): 2018–2028. 25891174 10.1056/NEJMoa1501824

[13] J. Larkin , V. Chiarion‐Sileni , R. Gonzalez , et al., “Combined Nivolumab and Ipilimumab or Monotherapy in Untreated Melanoma,” New England Journal of Medicine 373 (2015): 23–34. 26027431 10.1056/NEJMoa1504030 PMC5698905

[14] J. Aguiar , I. Santoro , H. Tadokoro , et al., “The Role of PD‐L1 Expression as a Predictive Biomarker in Advanced Non‐Small‐Cell Lung Cancer: A Network Meta‐Analysis,” Immunotherapy 8 (2016): 479–488. 26973128 10.2217/imt-2015-0002

[15] N. Rizvi , M. Hellmann , A. Snyder , et al., “Mutational Landscape Determines Sensitivity to PD‐1 Blockade in Non‐Small Cell Lung Cancer,” Science 348 (2015): 124–128. 25765070 10.1126/science.aaa1348 PMC4993154

[16] R. Herbst , P. Baas , D. Kim , et al., “Pembrolizumab Versus Docetaxel for Previously Treated, PD‐L1‐Positive, Advanced Non‐Small‐Cell Lung Cancer (KEYNOTE‐010): A Randomised Controlled Trial,” Lancet 387 (2016): 1540–1550. 26712084 10.1016/S0140-6736(15)01281-7

[17] M. Ayers , J. Lunceford , M. Nebozhyn , et al., “IFN‐γ–Related mRNA Profile Predicts Clinical Response to PD‐1 Blockade,” Journal of Clinical Investigation 127 (2017): 2930–2940. 28650338 10.1172/JCI91190 PMC5531419

[18] R. Bai , Z. Lv , D. Xu , and J. Cui , “Predictive Biomarkers for Cancer Immunotherapy With Immune Checkpoint Inhibitors,” Biomarker Research 8 (2020): 34. 32864131 10.1186/s40364-020-00209-0 PMC7450548

[19] T. Nishijima , H. Muss , S. Shachar , and S. Moschos , “Comparison of Efficacy of Immune Checkpoint Inhibitors (ICIs) Between Younger and Older Patients: A Systematic Review and Meta‐Analysis,” Cancer Treatment Reviews 45 (2016): 30–37. 26946217 10.1016/j.ctrv.2016.02.006

[20] C. Kugel, III , S. Douglass , M. Webster , et al., “Age Correlates With Response to Anti‐PD1, Reflecting Age‐Related Differences in Intratumoral Effector and Regulatory T‐Cell Populations,” Clinical Cancer Research 24 (2018): 5347–5356. 29898988 10.1158/1078-0432.CCR-18-1116 PMC6324578

[21] W. Murphy and D. Longo , “The Surprisingly Positive Association Between Obesity and Cancer Immunotherapy Efficacy,” JAMA 321 (2019): 1247–1248. 30882850 10.1001/jama.2019.0463

[22] Z. Wang , E. Aguilar , J. Luna , et al., “Paradoxical Effects of Obesity on T Cell Function During Tumor Progression and PD‐1 Checkpoint Blockade,” Nature Medicine 25 (2019): 141–151. 10.1038/s41591-018-0221-5 PMC6324991 30420753

[23] B. Guo and Y. Zang , “A Bayesian Phase I/II Biomarker‐Based Design for Identifying Subgroup‐Specific Optimal Dose for Immunotherapy,” Statistical Methods in Medical Research 31 (2022): 1104–1119. 35191780 10.1177/09622802221080753 PMC9305985

[24] B. Guo and Y. Zang , “BIPSE: A Biomarker‐Based Phase I/II Design for Immunotherapy Trials With Progression‐Free Survival Endpoint,” Statistics in Medicine 41 (2022): 1205–1224. 34821409 10.1002/sim.9265 PMC9335906

[25] J. Sokal , C. Aungst , M. Snyderman , and G. Gomez , “Immunotherapy of Chronic Myelocytic Leukemia: Effects of Different Vaccination Schedules,” International Conference on Immunotherapy of Cancer 277 (1976): 367–383. 10.1111/j.1749-6632.1976.tb41715.x 1069556

[26] A. Hejjaoui , H. Dhivert , F. Michal , and J. Bousquet , “Immunotherapy With a Standardized Dermatophagoides Pteronyssinus Extract: IV. Systemic Reactions According to the Immunotherapy Schedule,” Journal of Allergy and Clinical Immunology 85 (1996): 473–479. 10.1016/0091-6749(90)90157-y 2406324

[27] L. Cox , “Accelerated Immunotherapy Schedules: Review of Efficacy and Safety,” Annals of Allergy, Asthma, and Immunology 97 (2006): 126–138. 10.1016/S1081-1206(10)60003-8 16937741

[28] A. Tzeng , M. Kauke , E. F. Zhu , et al., “Temporally Programmed CD8α+ DC Activation Enhances Combination Cancer Immunotherapy,” Cell Reports 17 (2016): 2503–2511. 27926855 10.1016/j.celrep.2016.11.020 PMC5204262

[29] W. J. Lesterhuis , J. Haanen , and C. Punt , “Cancer Immunotherapy ‐ Revisited,” Nature Reviews Drug Discovery 10 (2011): 591–600. 21804596 10.1038/nrd3500

[30] T. M. Braun , P. Thall , H. nguyen , and M. DeLima , “Simultaneously Optimizing Dose and Schedule of a New Cytotoxic Agent,” Clinical Trials 4 (2007): 113–124. 17456511 10.1177/1740774507076934

[31] J. Zhang and T. M. Braun , “A Phase I Bayesian Adaptive Design to Simultaneously Optimize Dose and Schedule Assignments Both Between and Within Patients,” Journal of the American Statistical Association 108 (2013): 892–901. 10.1080/01621459.2013.806927 PMC3821780 24222927

[32] J. Lee , P. Thall , Y. Ji , and P. Mueller , “Bayesian Dose‐Finding in Two Treatment Cycles Based on the Joint Utility of Efficacy and Toxicity,” Journal of the American Statistical Association 110 (2015): 711–722. 26366026 10.1080/01621459.2014.926815 PMC4562700

[33] B. Guo , Y. Li , and Y. Yuan , “A Dose‐Schedule Finding Design for Phase I‐II Clinical Trials,” Journal of the Royal Statistical Society. Series C, Applied Statistics 65 (2016): 259–272. 26877554 10.1111/rssc.12113 PMC4747255

[34] R. Lin , P. Thall , and Y. Yuan , “An Adaptive Trial Design to Optimize Dose‐Schedule Regimes With Delayed Outcomes,” Biometrics 76 (2020): 304–315. 31273750 10.1111/biom.13116 PMC6942642

[35] R. Lin , P. Thall , and Y. Yuan , “A Phase I‐II Basket Trial Design to Optimize Dose‐Schedule Regimes Based on Delayed Outcomes,” Bayesian Analysis 16 (2021): 179–202. 34267857 10.1214/20-ba1205 PMC8277108

[36] M. De Lima , S. Giralt , P. Thall , et al., “Maintenance Therapy With Low‐Dose Azacitidine After Allogeneic Hematopoietic Stem Cell Transplantation for Recurrent Acute Myelogenous Leukemia or Myelodysplastic Syndrome: A Dose and Schedule Finding Study,” Cancer 116 (2010): 5420–5431. 20672358 10.1002/cncr.25500 PMC5669059

[37] C. Graux , A. Sonet , J. Maertens , et al., “A Phase I Dose‐Escalation Study of MSC1992371A, an Oral Inhibitor of Aurora and Other Kinases, in Advanced Hematologic Malignancies,” Leukemia Research 37 (2013): 1100–1106. 23746966 10.1016/j.leukres.2013.04.025

[38] N. Wages , J. O'Quigley , and M. Conaway , “Phase I Design for Completely or Partially Ordered Treatment Schedules,” Statistics in Medicine 33 (2014): 569–579. 24114957 10.1002/sim.5998 PMC3947103

[39] K. Cunanan and J. Koopmeiners , “Evaluating the Performance of Copula Models in Phase I‐II Clinical Trials Under Model Misspecification,” BMC Medical Research Methodology 14 (2014): 51. 24731155 10.1186/1471-2288-14-51 PMC4234400

[40] C. Cai , Y. Yuan , and Y. Ji , “A Bayesian Dose Finding Design for Oncology Clinical Trials of Combinational Biological Agents,” Journal of the Royal Statistical Society: Series C: Applied Statistics 63 (2014): 159–173. 24511160 10.1111/rssc.12039 PMC3913186

[41] A. Gelman , A. Jakulin , M. Pittau , and Y. Su , “A Weakly Informative Default Prior Distribution for Logistic and Other Regression Models,” 2008.

[42] S. Liu and V. Johnson , “A Robust Bayesian Dose‐Finding Design for Phase I/II Clinical Trials,” Biostatistics 17 (2016): 249–263. 26486139 10.1093/biostatistics/kxv040 PMC5006115

[43] E. J. Gumbel , “Bivariate Exponential Distributions,” Journal of the American Statistical Association 55 (1960): 698–707.

[44] Y. Cheung , “Simple Benchmark for Complex Dose Finding Studies,” Biometrics 70 (2014): 389–397. 24571185 10.1111/biom.12158 PMC4061271

[45] S. Liu , G. Yin , and Y. Yuan , “Bayesian Data Augmentation Dose Finding With Continual Reassessment Method and Delayed Toxicity,” Annals of Applied Statistics 4 (2013): 2138–2156. 10.1214/13-AOAS661 PMC3972824 24707327

[46] I. Jin , S. Liu , P. Thall , and Y. Yuan , “Using Data Augmentation to Facilitate Conduct of Phase I/II Clinical Trials With Delayed Outcomes,” Journal of the American Statistical Association 109 (2014): 525–536. 25382884 10.1080/01621459.2014.881740 PMC4217535

