# Supplementary material for: A Biomarker‐Based Dose–Schedule Optimization Design for Immunotherapy Trials
Source: Stat Med. 2026 Jan 22;45(1-2):e70357. doi: 10.1002/sim.70357 (PMC12828111; doi:10.1002/sim.70357)
Supplement: Supplementary file 1 — Data S1. Supporting Information. [file SIM-45-0-s001.pdf]

Supporting Information for “A Biomarker-Based Dose-Schedule Optimization Design for Immunotherapy Trials” by Y. Qiu, Y. Han, and B. Guo.

Table S1. True ordinal efficacy probabilities (q_0, q_1, q_2) under eight simulation scenarios, for each dose, schedule, and subgroup

**Scenario**		$M = 0$			$M = 1$		
		s_1	s_2	s_3	s_1	s_2	s_3
1	d_1	(0.55,0.35,0.1)	(0.55,0.35,0.1)	(0.55,0.35,0.1)	(0.48,0.39,0.13)	(0.48,0.39,0.13)	(0.48,0.39,0.13)
	d_2	(0.42,0.42,0.16)	(0.29,0.46,0.24)	(0.27,0.46,0.26)	(0.35,0.45,0.20)	(0.24,0.46,0.30)	(0.22,0.46,0.33)
	d_3	(0.39,0.43,0.17)	(0.26,0.46,0.28)	(0.24,0.46,0.30)	(0.33,0.46,0.22)	(0.21,0.45,0.34)	(0.19,0.44,0.37)
2	d_1	(0.67,0.23,0.10)	(0.67,0.23,0.10)	(0.67,0.23,0.10)	(0.62,0.26,0.12)	(0.62,0.26,0.12)	(0.62,0.26,0.12)
	d_2	(0.58,0.28,0.14)	(0.54,0.30,0.16)	(0.43,0.34,0.23)	(0.53,0.30,0.16)	(0.49,0.32,0.19)	(0.38,0.35,0.27)
	d_3	(0.54,0.30,0.16)	(0.48,0.33,0.20)	(0.32,0.36,0.32)	(0.49,0.32,0.19)	(0.43,0.34,0.23)	(0.28,0.36,0.36)
3	d_1	(0.93,0.05,0.02)	(0.89,0.08,0.03)	(0.89,0.08,0.03)	(0.92,0.06,0.02)	(0.87,0.09,0.04)	(0.87,0.09,0.04)
	d_2	(0.85,0.11,0.04)	(0.70,0.20,0.09)	(0.40,0.33,0.27)	(0.83,0.12,0.05)	(0.66,0.23,0.11)	(0.36,0.33,0.31)
	d_3	(0.77,0.16,0.07)	(0.50,0.30,0.20)	(0.11,0.22,0.67)	(0.73,0.19,0.08)	(0.45,0.32,0.23)	(0.09,0.20,0.72)
4	d_1	(0.65,0.17,0.18)	(0.48,0.21,0.31)	(0.65,0.17,0.18)	(0.60,0.19,0.21)	(0.43,0.22,0.35)	(0.60,0.19,0.21)
	d_2	(0.60,0.19,0.21)	(0.43,0.22,0.35)	(0.60,0.18,0.21)	(0.55,0.20,0.25)	(0.38,0.22,0.40)	(0.55,0.20,0.25)
	d_3	(0.60,0.19,0.21)	(0.43,0.22,0.35)	(0.60,0.19,0.21)	(0.55,0.20,0.25)	(0.38,0.22,0.40)	(10.55,0.20,0.25)
5	d_1	(0.71,0.15,0.14)	(0.60,0.19,0.21)	(0.69,0.16,0.15)	(0.67,0.16,0.17)	(0.55,0.20,0.25)	(0.65,0.17,0.18)
	d_2	(0.56,0.20,0.24)	(0.44,0.22,0.34)	(0.54,0.20,0.26)	(0.28,0.21,0.51)	(0.39,0.22,0.39)	(0.49,0.21,0.30)
	d_3	(0.56,0.20,0.24)	(0.43,0.22,0.35)	(0.53,0.20,0.26)	(0.51,0.21,0.28)	(0.39,0.22,0.39)	(0.48,0.21,0.30)
6	d_1	(0.80,0.11,0.09)	(0.88,0.07,0.05)	(0.87,0.07,0.06)	(0.77,0.12,0.11)	(0.86,0.08,0.06)	(0.85,0.08,0.07)
	d_2	(0.61,0.19,0.21)	(0.74,0.14,0.13)	(0.72,0.14,0.14)	(0.56,0.20,0.24)	(0.70,0.15,0.15)	(0.67,0.16,0.16)
	d_3	(0.47,0.22,0.31)	(0.62,0.18,0.20)	(0.60,0.19,0.21)	(0.46,0.22,0.32)	(0.57,0.19,0.23)	(0.29,0.21,0.50)
7	d_1	(0.82,0.06,0.12)	(0.73,0.09,0.18)	(0.80,0.07,0.13)	(0.79,0.07,0.14)	(0.45,0.12,0.43)	(0.77,0.08,0.15)
	d_2	(0.75,0.08,0.17)	(0.65,0.10,0.25)	(0.72,0.09,0.19)	(0.71,0.09,0.19)	(0.60,0.11,0.29)	(0.67,0.10,0.23)
	d_3	(0.73,0.09,0.18)	(0.63,0.11,0.27)	(0.69,0.10,0.21)	(0.69,0.10,0.21)	(0.63,0.12,0.25)	(0.64,0.10,0.25)
8	d_1	(0.67,0.10,0.23)	(0.69,0.1,0.21)	(0.71,0.10,0.20)	(0.62,0.11,0.27)	(0.65,0.10,0.25)	(0.67,0.10,0.23)
	d_2	(0.58,0.12,0.30)	(0.60,0.11,0.29)	(0.62,0.11,0.27)	(0.53,0.12,0.36)	(0.55,0.12,0.33)	(0.58,0.12,0.30)
	d_3	(0.56,0.12,0.32)	(0.59,0.11,0.30)	(0.61,0.11,0.28)	(0.51,0.12,0.37)	(0.54,0.12,0.34)	(0.56,0.12,0.32)

Table S2. Sensitivity analysis: selection percentage and average number of patients treated at each dose-schedule combination. Proposed(0.3) and Proposed(0.7) indicate the cases when marker-positive prevalence is 0.3 and 0.7 respectively.

**Design**		$M=0$			$M=1$		
		s_1	s_2	s_3	s_1	s_2	s_3
				Scenario 1			
Proposed	d_1	6.1(10.7)	6.0(11.0)	0.6(7.7)	7.0(11.1)	6.2(11.0)	0.7(7.7)
	d_2	22.6(11.5)	**61.9(11.6)**	0.4(2.1)	23.0(11.3)	**60.1(11.2)**	0.4(2.1)
	d_3	1.7(2.5)	0.7(1.9)	0.0(1.1)	2.1(2.4)	0.5(1.9)	0.0(1.2)
Proposed(0.3)	d_1	6.5(15.5)	4.5(15.0)	2.5(10.7)	7.6(6.8)	4.9(6.6)	2.8(4.9)
	d_2	24.7(16.2)	**60.0(15.9)**	0.0(3.0)	25.0(6.7)	**57.9(6.4)**	0.0(1.2)
	d_3	0.9(3.4)	0.9(2.6)	0.0(1.7)	0.8(1.5)	1.0(1.2)	0.0(0.7)
Proposed(0.7)	d_1	6.2(6.8)	6.3(6.7)	1.1(4.6)	6.6(15.3)	6.4(15.4)	1.3(11.5)
	d_2	25.4(6.8)	**58.8(6.7)**	0.1(1.2)	26.8(15.9)	**56.4(15.6)**	0.2(2.8)
	d_3	1.1(1.4)	1.0(1.1)	0.0(0.7)	1.3(3.4)	1.0(2.7)	0.0(1.6)
				Scenario 2			
Proposed	d_1	3.8(7.3)	4.2(7.6)	2.7(7.6)	4.8(8.2)	4.6(8.0)	2.6(8.0)
	d_2	9.1(8.7)	15.0(9.4)	**60.9(11.8)**	8.5(8.8)	14.6(9.1)	**60.2(11.0)**
	d_3	1.6(2.9)	1.3(2.3)	1.4(2.1)	2.0(2.8)	1.3(2.3)	1.4(2.0)
Proposed(0.3)	d_1	4.7(10.8)	3.9(11.0)	3.4(11.1)	5.3(4.9)	5.6(4.7)	3.7(5.2)
	d_2	10.2(12.1)	15.8(13.0)	**58.7(15.9)**	11.0(5.2)	15.4(5.5)	**55.5(6.4)**
	d_3	1.5(3.9)	1.0(3.2)	0.8(3.0)	1.7(1.6)	0.9(1.4)	0.9(1.2)
Proposed(0.7)	d_1	5.5(4.7)	3.8(4.6)	3.8(5.0)	5.0(11.8)	4.0(11.4)	3.5(11.9)
	d_2	10.5(5.2)	13.6(5.4)	**58.1(6.8)**	11.6(12.1)	15.1(12.5)	**56.6(14.4)**
	d_3	1.9(1.7)	1.5(1.4)	1.2(1.2)	1.6(3.9)	1.5(3.1)	1.1(2.9)
				Scenario 3			
Proposed	d_1	0.0(1.7)	0.1(2.3)	0.1(3.5)	0.0(2.4)	0.2(4.5)	0.1(5.5)
	d_2	0.1(2.4)	13.8(14.1)	0.8(4.1)	0.8(4.7)	18.8(15.1)	0.3(3.0)
	d_3	2.6(4.8)	**66.1(19.9)**	0.0(1.3)	4.2(6.1)	**63.8(13.8**)	0.1(1.3)
Proposed(0.3)	d_1	0.0(2.4)	0.1(3.7)	0.0(5.0)	0.0(1.6)	0.2(3.4)	0.1(3.7)
	d_2	0.3(3.5)	15.8(21.2)	1.0(5.4)	0.6(2.9)	15.8(8.9)	0.4(1.6)
	d_3	2.3(7.0)	**65.1(26.0)**	0.0(1.8)	4.0(3.9)	**65.9(7.5)**)	0.1(0.8)
Proposed(0.7)	d_1	0.0(1.0)	0.2(1.4)	0.4(2.1)	0.0(3.2)	0.4(5.7)	0.2(7.1)
	d_2	0.0(1.3)	11.0(7.9)	1.5(2.6)	0.3(5.5)	18.1(21.1)	0.7(4.5)
	d_3	2.2(2.8)	**66.2(12.2)**	0.0(0.9)	3.5(8.5)	**62.7(20.4**)	0.0(1.8)
				Scenario 4			
Proposed	d_1	6.1(11.2)	**81.0(20.9)**	1.6(8.4)	7.0(12.4)	**79.6(18.7)**	2.1(9.2)
	d_2	5.1(8.3)	6.2(5.4)	0.0(1.9)	5.8(8.4)	5.5(4.9)	0.0(1.9)
	d_3	0.0(1.6)	0.0(1.4)	0.0(1.2)	0.0(1.6)	0.0(1.4)	0.0(1.2)
Proposed(0.3)	d_1	6.1(15.1)	**79.3(28.1)**	1.9(12.2)	6.7(7.2)	**77.1(11.1)**	1.8(5.8)
	d_2	4.9(11.9)	7.7(7.9)	0.0(2.8)	6.9(5.1)	7.5(3.2)	0.0(1.2)
	d_3	0.0(2.3)	0.0(1.9)	0.0(1.7)	0.0(1.0)	0.0(0.9)	0.0(0.7)
Proposed(0.7)	d_1	6.3(6.6)	**80.0(12.6)**	1.5(5.1)	6.3(16.3)	**79.8(26.7)**	1.9(13.2)
	d_2	6.4(5.0)	5.7(3.3)	0.0(1.1)	6.5(11.7)	5.4(7.4)	0.0(2.6)
	d_3	0.0(1.0)	0.0(0.9)	0.0(0.8)	0.0(2.2)	0.0(1.9)	0.0(1.6)

Table S2.(continued)

**Design**		$M = 0$			$M = 1$		
		s_1	s_2	s_3	s_1	s_2	s_3
				Scenario 5			
Proposed	d_1	1.0(6.9)	9.1(10.2)	0.7(5.6)	0.7(8.3)	7.2(10.6)	0.5(6.7)
	d_2	32.8(14.2)	**54.1(14.1)**	0.6(2.9)	**53.4(13.6)**	36.6(12.4)	0.5(2.9)
	d_3	0.8(2.5)	0.9(2.1)	0.0(1.3)	0.3(2.4)	0.8(2.1)	0.0(1.3)
Proposed(0.3)	d_1	0.6(10.5)	11.7(16.4)	0.9(8.5)	1.4(5.3)	8.8(6.9)	0.4(4.3)
	d_2	27.2(17.5)	**58.0(18.9)**	0.2(3.9)	**54.6(7.1)**	33.3(7.4)	0.3(1.7)
	d_3	0.6(3.4)	0.7(2.9)	0.0(1.9)	0.5(1.4)	0.7(1.2)	0.0(0.8)
Proposed(0.7)	d_1	1.0(4.5)	6.8(6.2)	0.9(3.5)	0.8(12.1)	5.4(14.8)	0.5(9.5)
	d_2	38.3(8.4)	**51.4(8.1)**	0.3(1.7)	**63.1(19.3)**	29.7(16.5)	0.0(3.8)
	d_3	0.4(1.5)	0.9(1.3)	0.0(0.8)	0.1(3.5)	0.4(2.8)	0.0(1.9)
				Scenario 6			
Proposed	d_1	0.5(3.4)	0.0(2.0)	0.1(2.3)	0.5(4.6)	0.0(2.7)	0.1(3.5)
	d_2	4.9(9.3)	0.4(4.8)	0.4(6.8)	6.0(8.8)	0.8(5.7)	0.7(7.3)
	d_3	**53.9(12.6)**	5.2(7.7)	33.3(10.8)	34.2(10.1)	4.2(7.2)	**52.8(9.7)**
Proposed(0.3)	d_1	0.1(5.3)	0.0(3.0)	0.0(3.5)	0.2(3.2)	0.2(1.9)	0.1(2.3)
	d_2	5.8(13.7)	0.2(6.7)	0.7(9.0)	6.6(5.4)	0.5(3.5)	0.7(4.1)
	d_3	**58.1(17.5)**	9.9(10.6)	24.1(13.9)	35.7(6.0)	7.9(4.3)	**47.3(5.2)**
Proposed(0.7)	d_1	0.4(2.1)	0.0(1.2)	0.1(1.4)	0.7(6.5)	0.1(3.9)	0.0(4.8)
	d_2	5.7(5.4)	0.3(2.7)	0.6(4.3)	6.6(12.4)	0.4(7.9)	0.8(9.9)
	d_3	**53.3(7.1)**	7.4(4.5)	30.6(6.7)	26.2(13.8)	5.0(9.9)	**59.4(14.7)**
				Scenario 7			
Proposed	d_1	0.6(3.5)	37.5(16.4)	0.9(4.4)	1.0(5.2)	**66.5(17.1)**	0.5(5.9)
	d_2	5.6(6.2)	**43.5(15.3)**	0.9(3.0)	5.2(7.6)	20.5(13.1)	0.4(2.8)
	d_3	2.2(3.5)	3.2(3.3)	0.0(1.4)	1.8(3.2)	1.3(3.0)	0.0(1.4)
Proposed(0.3)	d_1	1.7(5.2)	28.8(19.3)	1.7(6.3)	2.6(3.5)	**60.8(9.2)**	2.3(3.8)
	d_2	5.7(9.1)	**43.0(23.3)**	1.0(4.5)	7.0(4.7)	20.7(8.2)	0.3(1.7)
	d_3	2.9(4.7)	1.5(4.5)	0.0(1.9)	2.5(1.9)	0.8(1.7)	0.0(0.8)
Proposed(0.7)	d_1	1.2(2.3)	31.7(11.2)	0.9(2.5)	1.2(7.7)	**73.3(24.9)**	1.0(8.3)
	d_2	3.2(3.4)	**47.4(8.7)**	1.1(1.7)	3.7(10.4)	14.3(17.7)	0.4(4.0)
	d_3	2.9(2.1)	2.3(1.9)	0.0(0.8)	1.7(4.4)	1.2(3.7)	0.0(1.9)
				Scenario 8			
Proposed	d_1	10.4(8.6)	6.9(7.4)	1.0(4.7)	12.3(10.1)	6.3(8.3)	0.9(5.6)
	d_2	**45.7(16.5)**	5.8(6.5)	0.1(2.2)	**44.1(14.8)**	5.7(5.9)	0.1(2.2)
	d_3	**28.9(10.8)**	0.2(1.9)	0.0(1.2)	**29.7(9.8)**	0.2(1.8)	0.0(1.2)
Proposed(0.3)	d_1	9.5(12.1)	5.6(10.3)	2.0(7.4)	11.1(6.0)	7.8(5.1)	1.3(3.7)
	d_2	**42.0(22.1)**	6.3(8.9)	0.1(3.2)	**40.1(8.3)**	5.5(3.8)	0.1(1.5)
	d_3	**32.3(14.9)**	0.1(2.7)	0.0(1.7)	**32.8(5.6)**	0.3(1.1)	0.0(0.7)
Proposed(0.7)	d_1	10.2(5.4)	5.6(4.3)	1.0(3.0)	14.0(14.1)	5.5(11.7)	1.0(8.2)
	d_2	**45.7(9.1)**)	4.8(4.0)	0.2(1.4)	**44.3(19.8)**	5.2(9.0)	0.1(3.3)
	d_3	**29.6(6.2)**	0.5(1.2)	0.0(0.7)	**28.5(13.2)**	0.2(2.6)	0.0(1.7)

Table S3 .Sensitivity analysis: selection percentage and average number of patients treated at each dose-schedule combination. Proposed(150) represents the cases with a maximum sample size 150.

**Design**		$M=0$			$M=1$		
		s_1	s_2	s_3	s_1	s_2	s_3
				Scenario 1			
Proposed	d_1	6.1(10.7)	6.0(11.0)	0.6(7.7)	7.0(11.1)	6.2(11.0)	0.7(7.7)
	d_2	22.6(11.5)	**61.9(11.6)**	0.4(2.1)	23.0(11.3)	**60.1(11.2)**	0.4(2.1)
	d_3	1.7(2.5)	0.7(1.9)	0.0(1.1)	2.1(2.4)	0.5(1.9)	0.0(1.2)
Proposed(150)	d_1	5.9(14.2)	4.7(14.1)	1.3(8.9)	7.5(14.3)	4.5(14.1)	1.0(8.9)
	d_2	21.1(14.4)	**64.7(15.5)**	0.3(2.1)	21.2(14.6)	**62.9(14.8)**	0.2(2.0)
	d_3	1.4(2.6)	0.6(2.0)	0.0(1.2)	2.2(2.6)	0.5(1.9)	0.0(1.1)
				Scenario 2			
Proposed	d_1	3.8(7.3)	4.2(7.6)	2.7(7.6)	4.8(8.2)	4.6(8.0)	2.6(8.0)
	d_2	9.1(8.7)	15.0(9.4)	**60.9(11.8)**	8.5(8.8)	14.6(9.1)	**60.2(11.0)**
	d_3	1.6(2.9)	1.3(2.3)	1.4(2.1)	2.0(2.8)	1.3(2.3)	1.4(2.0)
Proposed(150)	d_1	4.0(9.6)	3.8(9.4)	3.8(10.8)	4.2(10.6)	4.1(10.1)	4.1(11.0)
	d_2	8.2(10.8)	14.1(11.6)	**63.5(15.4)**	8.5(10.8)	14.2(11.5)	**62.0(13.7)**
	d_3	1.0(2.9)	0.7(2.4)	0.9(2.1)	1.0(2.9)	1.0(2.3)	0.9(2.0)
				Scenario 3			
Proposed	d_1	0.0(1.7)	0.1(2.3)	0.1(3.5)	0.0(2.4)	0.2(4.5)	0.1(5.5)
	d_2	0.1(2.4)	13.8(14.1)	0.8(4.1)	0.8(4.7)	18.8(15.1)	0.3(3.0)
	d_3	2.6(4.8)	**66.1(19.9)**	0.0(1.3)	4.2(6.1)	**63.8(13.8)**	0.1(1.3)
Proposed(150)	d_1	0.0(1.7)	0.0(2.3)	0.2(3.5)	0.0(2.5)	0.0(5.1)	0.1(5.9)
	d_2	0.0(2.5)	10.8(17.5)	0.6(4.0)	0.6(5.1)	17.7(20.0)	0.3(2.8)
	d_3	1.0(5.5)	**69.1(27.4)**	0.0(1.4)	3.1(8.1)	**65.8(18.9)**	0.0(1.3)
				Scenario 4			
Proposed	d_1	6.1(11.2)	**81.0(20.9)**	1.6(8.4)	7.0(12.4)	**79.6(18.7)**	2.1(9.2)
	d_2	5.1(8.3)	6.2(5.4)	0.0(1.9)	5.8(8.4)	5.5(4.9)	0.0(1.9)
	d_3	0.0(1.6)	0.0(1.4)	0.0(1.2)	0.0(1.6)	0.0(1.4)	0.0(1.2)
Proposed(150)	d_1	3.9(13.6)	**84.0(28.2)**	1.1(10.5)	4.4(15.6)	**83.5(25.6)**	1.2(11.2)
	d_2	5.2(10.5)	5.8(6.0)	0.0(1.9)	5.2(11.1)	5.7(5.6)	0.0(1.9)
	d_3	0.0(1.6)	0.0(1.4)	0.0(1.2)	0.0(1.6)	0.0(1.4)	0.0(1.2)

Table S3 (continued)

**Design**		$M = 0$			$M = 1$		
		s_1	s_2	s_3	s_1	s_2	s_3
		Scenario 5					
Proposed	d_1	1.0(6.9)	9.1(10.2)	0.7(5.6)	0.7(8.3)	7.2(10.6)	0.5(6.7)
	d_2	32.8(14.2)	**54.1(14.1)**	0.6(2.9)	**53.4(13.6)**	36.6(12.4)	0.5(2.9)
	d_3	0.8(2.5)	0.9(2.1)	0.0(1.3)	0.3(2.4)	0.8(2.1)	0.0(1.3)
Proposed(150)	d_1	1.3(8.9)	8.3(13.8)	0.9(7.1)	0.9(10.3)	5.2(14.1)	0.3(8.3)
	d_2	29.8(17.8)	**58.1(18.4)**	0.1(2.9)	**59.7(17.4)**	32.6(16.0)	0.0(2.6)
	d_3	0.8(2.8)	0.6(2.2)	0.0(1.3)	0.5(2.6)	0.7(2.1)	0.0(1.3)
		Scenario 6					
Proposed	d_1	0.5(3.4)	0.0(2.0)	0.1(2.3)	0.5(4.6)	0.0(2.7)	0.1(3.5)
	d_2	4.9(9.3)	0.4(4.8)	0.4(6.8)	6.0(8.8)	0.8(5.7)	0.7(7.3)
	d_3	**53.9(12.6)**	5.2(7.7)	33.3(10.8)	34.2(10.1)	4.2(7.2)	**52.8(9.7)**
Proposed(150)	d_1	0.1(4.2)	0.0(2.0)	0.0(2.4)	0.2(6.0)	0.0(3.2)	0.0(3.9)
	d_2	4.8(12.5)	0.0(5.5)	0.7(8.3)	6.0(11.0)	0.0(7.1)	0.5(8.7)
	d_3	**57.0(16.1)**	6.3(9.6)	30.4(14.0)	34.3(13.0)	4.4(9.2)	**54.3(12.8)**
		Scenario 7					
Proposed	d_1	0.6(3.5)	37.5(16.4)	0.9(4.4)	1.0(5.2)	**66.5(17.1)**	0.5(5.9)
	d_2	5.6(6.2)	**43.5(15.3)**	0.9(3.0)	5.2(7.6)	20.5(13.1)	0.4(2.8)
	d_3	2.2(3.5)	3.2(3.3)	0.0(1.4)	1.8(3.2)	1.3(3.0)	0.0(1.4)
Proposed(150)	d_1	0.0(3.8)	30.9(21.6)	0.5(4.8)	0.9(6.1)	**71.4(23.6)**	0.3(6.7)
	d_2	3.2(6.6)	**47.2(20.6)**	0.5(3.2)	4.6(9.2)	17.1(16.6)	0.1(3.0)
	d_3	1.9(3.9)	1.5(3.6)	0.0(1.4)	1.1(3.6)	1.0(3.0)	0.0(1.3)
		Scenario 8					
Proposed	d_1	10.4(8.6)	6.9(7.4)	1.0(4.7)	12.3(10.1)	6.3(8.3)	0.9(5.6)
	d_2	**45.7(16.5)**	5.8(6.5)	0.1(2.2)	**44.1(14.8)**	5.7(5.9)	0.1(2.2)
	d_3	**28.9(10.8)**	0.2(1.9)	0.0(1.2)	**29.7(9.8)**	0.2(1.8)	0.0(1.2)
Proposed(150)	d_1	7.8(10.9)	3.9(9.2)	0.8(5.9)	8.6(12.9)	5.5(10.8)	0.8(6.7)
	d_2	**46.9(20.3)**	5.1(7.7)	0.0(2.4)	**46.1(18.4)**	4.4(7.0)	0.1(2.3)
	d_3	**33.6(14.6)**	0.2(2.0)	0.0(1.2)	**33.3(13.4)**	0.3(1.9)	0.0(1.2)

Table S4 .Sensitivity analysis: selection percentage and average number of patients treated at each dose-schedule combination. Proposed(non-informative priors) represents the cases with specificed non-informative priors.

**Design**		$M = 0$			$M = 1$		
		s_1	s_2	s_3	s_1	s_2	s_3
				Scenario 1			
Proposed	d_1	6.1(10.7)	6.0(11.0)	0.6(7.7)	7.0(11.1)	6.2(11.0)	0.7(7.7)
	d_2	22.6(11.5)	**61.9(11.6)**	0.4(2.1)	23.0(11.3)	**60.1(11.2)**	0.4(2.1)
	d_3	1.7(2.5)	0.7(1.9)	0.0(1.1)	2.1(2.4)	0.5(1.9)	0.0(1.2)
Proposed	d_1	5.6(10.3)	7.0(10.6)	2.7(9.3)	7.2(10.9)	6.3(10.7)	3.2(9.4)
(non-informative	d_2	20.0(10.5)	**61.7(11.7)**	2.3(2.6)	20.5(10.7)	**60.0(11.4)**	1.5(2.5)
priors)	d_3	0.4(1.9)	0.3(1.6)	0.0(1.2)	0.9(1.9)	0.4(1.7)	0.0(1.2)
				Scenario 2			
Proposed	d_1	3.8(7.3)	4.2(7.6)	2.7(7.6)	4.8(8.2)	4.6(8.0)	2.6(8.0)
	d_2	9.1(8.7)	15.0(9.4)	**60.9(11.8)**	8.5(8.8)	14.6(9.1)	**60.2(11.0)**
	d_3	1.6(2.9)	1.3(2.3)	1.4(2.1)	2.0(2.8)	1.3(2.3)	1.4(2.0)
Proposed	d_1	3.6(7.3)	2.7(7.4)	3.6(7.9)	4.2(8.0)	2.9(8.0)	4.8(8.3)
(non-informative	d_2	7.7(8.6)	13.3(9.7)	**60.1(11.7)**	9.2(8.8)	13.9(9.3)	**60.7(11.5)**
priors)	d_3	3.0(2.3)	2.5(2.1)	1.5(1.9)	2.3(2.5)	1.5(2.0)	0.5(1.9)
				Scenario 3			
Proposed	d_1	0.0(1.7)	0.1(2.3)	0.1(3.5)	0.0(2.4)	0.2(4.5)	0.1(5.5)
	d_2	0.1(2.4)	13.8(14.1)	0.8(4.1)	0.8(4.7)	18.8(15.1)	0.3(3.0)
	d_3	2.6(4.8)	**66.1(19.9)**	0.0(1.3)	4.2(6.1)	**63.8(13.8**)	0.1(1.3)
Proposed	d_1	0.0(1.7)	0.0(2.2)	0.1(3.4)	0.1(2.3)	0.4(4.3)	0.5(5.9)
(non-informative	d_2	0.1(2.4)	20.7(15.5)	2.2(6.8)	1.0(4.8)	30.7(17.3)	0.7(4.5)
priors)	d_3	3.1(4.7)	**49.8(14.5)**	0.0(1.3)	5.3(5.5)	**48.2(10.1**)	0.0(1.4)
				Scenario 4			
Proposed	d_1	6.1(11.2)	**81.0(20.9)**	1.6(8.4)	7.0(12.4)	**79.6(18.7)**	2.1(9.2)
	d_2	5.1(8.3)	6.2(5.4)	0.0(1.9)	5.8(8.4)	5.5(4.9)	0.0(1.9)
	d_3	0.0(1.6)	0.0(1.4)	0.0(1.2)	0.0(1.6)	0.0(1.4)	0.0(1.2)
Proposed	d_1	6.6(10.7)	**78.0(20.1)**	2.8(9.3)	6.9(11.5)	**78.1(18.3)**	3.1(10.3)
(non-informative	d_2	6.5(8.4)	6.0(5.7)	0.0(2.0)	6.6(8.2)	5.3(5.3)	0.0(2.2)
priors)	d_3	0.0(1.5)	0.0(1.4)	0.0(1.1)	0.0(1.5)	0.0(1.4)	0.0(1.2)

Table S4 (continued)

**Design**		$M = 0$			$M = 1$		
		s_1	s_2	s_3	s_1	s_2	s_3
		Scenario 5					
Proposed	d_1	1.0(6.9)	9.1(10.2)	0.7(5.6)	0.7(8.3)	7.2(10.6)	0.5(6.7)
	d_2	32.8(14.2)	**54.1(14.1)**	0.6(2.9)	**53.4(13.6)**	36.6(12.4)	0.5(2.9)
	d_3	0.8(2.5)	0.9(2.1)	0.0(1.3)	0.3(2.4)	0.8(2.1)	0.0(1.3)
Proposed	d_1	1.3(7.2)	9.7(10.6)	0.5(6.0)	1.3(8.5)	7.8(10.6)	0.4(7.0)
(non-informative	d_2	34.4(13.6)	**53.2(14.2)**	0.3(3.6)	**53.1(13.0)**	36.5(12.5)	0.3(3.5)
priors)	d_3	0.3(1.9)	0.3(1.7)	0.0(1.3)	0.3(1.9)	0.3(1.7)	0.0(1.3)
		Scenario 6					
Proposed	d_1	0.5(3.4)	0.0(2.0)	0.1(2.3)	0.5(4.6)	0.0(2.7)	0.1(3.5)
	d_2	4.9(9.3)	0.4(4.8)	0.4(6.8)	6.0(8.8)	0.8(5.7)	0.7(7.3)
	d_3	**53.9(12.6)**	5.2(7.7)	33.3(10.8)	34.2(10.1)	4.2(7.2)	**52.8(9.7)**
Proposed	d_1	0.3(3.4)	0.0(2.0)	0.0(2.4)	0.5(4.7)	0.0(2.9)	0.0(3.5)
(non-informative	d_2	5.8(9.7)	0.6(4.9)	0.5(6.8)	8.0(9.0)	0.6(5.8)	0.1(7.2)
priors)	d_3	**53.3(12.5)**	7.3(7.5)	31.7(10.6)	35.5(9.9)	4.6(7.1)	**50.3(9.7)**
		Scenario 7					
Proposed	d_1	0.6(3.5)	37.5(16.4)	0.9(4.4)	1.0(5.2)	**66.5(17.1)**	0.5(5.9)
	d_2	5.6(6.2)	**43.5(15.3)**	0.9(3.0)	5.2(7.6)	20.5(13.1)	0.4(2.8)
	d_3	2.2(3.5)	3.2(3.3)	0.0(1.4)	1.8(3.2)	1.3(3.0)	0.0(1.4)
Proposed	d_1	0.8(3.8)	39.8(16.2)	1.4(4.7)	1.5(5.9)	**65.4(16.5)**	1.2(6.6)
(non-informative	d_2	5.6(6.5)	**36.5(15.4)**	0.9(3.9)	5.8(7.8)	20.9(13.2)	0.7(3.6)
priors)	d_3	1.0(2.4)	0.9(2.3)	0.0(1.4)	0.5(2.1)	0.6(2.1)	0.0(1.3)
		Scenario 8					
Proposed	d_1	10.4(8.6)	6.9(7.4)	1.0(4.7)	12.3(10.1)	6.3(8.3)	0.9(5.6)
	d_2	**45.7(16.5**)	5.8(6.5)	0.1(2.2)	**44.1(14.8)**	5.7(5.9)	0.1(2.2)
	d_3	**28.9(10.8)**	0.2(1.9)	0.0(1.2)	**29.7(9.8)**	0.2(1.8)	0.0(1.2)
Proposed	d_1	10.1(9.0)	7.9(7.7)	2.9(6.3)	13.8(10.2)	8.5(8.8)	2.4(7.4)
(non-informative	d_2	**51.0(16.5**)	8.0(7.7)	0.0(3.0)	**49.0(14.6)**	7.5(7.1)	0.1(2.9)
priors)	d_3	**18.0(6.6)**	0.2(1.7)	0.0(1.2)	**17.9(5.8)**	0.2(1.7)	0.0(1.2)

Benchmark method

To obtain benchmark performances for our dose–schedule objective given a total sample size N (with the planned subgroup split), we applied a nonparametric full-information (oracle) benchmark for dose-finding trials (Cheung, 2014). In our setting with patients' biomarker information, the benchmark constructs complete outcome information for each biomarker subgroup (e.g., 60 patients per subgroup in the main simulation study) across all dose–schedule combinations and then evaluates the same decision rule as the trial.

To ensure consistency of outcomes across cells, we generated complete outcomes using *latent variables* that are shared for each patient across all dose–schedule combinations. With these complete profiles, we computed nonparametric marginal estimates by dose–schedule cell and subgroup M:

$$\hat{p}_{jk} = \widehat{\Pr}(\text{DLT at } (d_j, s_k)), \qquad \hat{q}_{\ell,jk} = \widehat{\Pr}(Y = \ell \text{ at } (d_j, s_k)), \quad \ell \in \{0, 1, 2\},$$

and the estimated response probability $\hat{\pi}_{jk} = 1 - \hat{q}_{0,jk}$. For each subgroup, we then applied the same admissibility rules as in the trial,

$$\mathcal{A}_M = \big\{(j,k) : \ \hat{p}_{jk} < \phi_T \ \text{ and } \ \hat{\pi}_{jk} > \phi_E\big\},$$

and the same utility,

$$U_{jk} = \hat{q}_{2,jk} + w_1\, \hat{q}_{1,jk} - w_2\, \hat{p}_{jk} - w_3\, \hat{p}_{jk}\, I\{\hat{p}_{jk} > \phi_T\},$$

selecting the admissible dose–schedule combination with maximal utility.

Repeating this procedure over 5,000 simulated trials yielded the benchmark selection proportions for each (d_j, s_k) within each subgroup. The results are summarized in Table S5.

Table S5. Sensitivity analysis: percentage of correct selection (PCS) of optimal dose-schedule combination for the proposed designs compared to benchmark performance.

Scenario	Design	PCS of optimal dose-schedule combination	
		$M = 0$	$M = 1$
1	Proposed	61.9	60.1
	Alternative 1	43.3	43.0
	Alternative 2	53.0	57.3
	Benchmark	98.2	98.6
2	Proposed	60.9	60.2
	Alternative 1	36.8	37.4
	Alternative 2	47.7	54.4
	Benchmark	99.1	98.9
3	Proposed	66.1	63.8
	Alternative 1	54.0	55.2
	Alternative 2	49.1	65.8
	Benchmark	98.3	98.3
4	Proposed	81.0	79.6
	Alternative 1	76.8	76.4
	Alternative 2	81.5	81.2
	Benchmark	99.7	99.8
5	Proposed	54.1	53.4
	Alternative 1	49.6	52.1
	Alternative 2	56.0	32.2
	Benchmark	98.5	99.6
6	Proposed	53.9	52.8
	Alternative 1	52.4	50.5
	Alternative 2	62.8	34.2
	Benchmark	99.9	99.7
7	Proposed	43.5	66.5
	Alternative 1	33.1	68.6
	Alternative 2	32.8	51.2
	Benchmark	82.4	99.8
8	Proposed	74.6	73.8
	Alternative 1	74.7	70.4
	Alternative 2	52.9	60.0
	Benchmark	99.8	99.8

Table S6. True immune response mean, toxicity and efficacy probabilities and utilities $\begin{bmatrix} \mu_Z, & p \\ \pi, & U_{true} \end{bmatrix}$ under additional simulation scenarios, for each dose, schedule, and subgroup

	$M=0$					$M=1$				
	s_1	s_2	s_3	s_4	s_5	s_1	s_2	s_3	s_4	s_5
Scenario 1										
d_1	1.15, 0.10	5.73, 0.16	11.45, 0.43			1.40, 0.10	6.99, 0.16	13.99, 0.43		
	0.27, 0.13	0.27, 0.11	0.27, −1.17			0.33, 0.17	0.33, 0.15	0.33, −1.13		
d_2	1.97, 0.14	**9.86, 0.18**	19.71, 0.55			2.41, 0.14	**12.04, 0.18**	24.08, 0.55		
	0.32, 0.15	**0.56, 0.30**	0.81, −0.77			0.39, 0.19	**0.63, 0.36**	0.86, −0.71		
d_3	2.00, 0.48	10.00, 0.55	19.99, 0.65			2.44, 0.48	12.21, 0.55	24.42, 0.65		
	0.32, −1.15	0.57, −1.00	0.83, −0.79			0.39, −1.11	0.64, −0.95	0.87, −0.73		
d_4	2.00, 0.55	10.00, 0.60	20.00, 0.68			2.44, 0.55	12.21, 0.60	24.43, 0.68		
	0.32, −1.17	0.57, −1.02	0.83, −0.79			0.39, −1.13	0.64, −0.96	0.87, −0.74		
Scenario 2										
d_1	2.07, 0.05	3.11, 0.08	5.70, 0.10			3.09, 0.05	4.64, 0.08	8.51, 0.10		
	0.23, 0.13	0.23, 0.12	0.23, 0.12			0.27, 0.16	0.27, 0.15	0.27, 0.14		
d_2	6.21, 0.12	9.32, 0.14	**17.09, 0.15**			9.27, 0.12	13.91, 0.14	**25.50, 0.15**		
	0.31, 0.17	0.36, 0.19	**0.48, 0.28**			0.36, 0.20	0.41, 0.23	**0.53, 0.32**		
d_3	7.46, 0.34	11.19, 0.39	20.52, 0.45			11.13, 0.34	16.70, 0.39	30.61, 0.45		
	0.34, −1.08	0.40, −1.05	0.30, −1.14			0.39, −1.05	0.45, −1.01	0.30, −1.14		
d_4	7.84, 0.40	11.76, 0.47	21.55, 0.52			11.69, 0.40	17.54, 0.47	32.16, 0.52		
	0.35, −1.09	0.42, −1.06	0.15, −1.26			0.40, −1.06	0.47, −1.03	0.15, −1.26		
Scenario 3										
d_1	4.58, 0.10	6.87, 0.16	8.59, 0.43			5.59, 0.10	8.39, 0.16	10.49, 0.43		
	0.17, 0.07	0.23, 0.09	0.21, −1.20			0.21, 0.09	0.29, 0.12	0.27, −1.17		
d_2	7.89, 0.14	**11.83, 0.18**	14.79, 0.55			9.63, 0.14	**14.45, 0.18**	18.06, 0.55		
	0.28, 0.12	**0.45, 0.22**	0.48, −1.07			0.35, 0.16	**0.52, 0.27**	0.56, −1.01		
d_3	8.00, 0.38	11.99, 0.40	14.99, 0.60			9.77, 0.38	14.65, 0.40	18.31, 0.60		
	0.29, −1.15	0.46, −1.04	0.50, −1.07			0.35, −1.10	0.53, −0.99	0.57, −1.02		
d_4	8.00, 0.48	12.00, 0.55	15.00, 0.65			9.77, 0.48	14.66, 0.55	18.32, 0.65		
	0.29, −1.18	0.46, −1.09	0.50, −1.09			0.35, −1.13	0.53, −1.03	0.57, −1.03		
d_5	8.00, 0.55	12.00, 0.60	15.00, 0.68			9.77, 0.55	14.66, 0.60	18.32, 0.68		
	0.29, −1.20	0.46, −1.10	0.50, −1.10			0.35, −1.16	0.53, −1.05	0.57, −1.04		
Scenario 4										
d_1	1.11, 0.05	1.67, 0.06	2.79, 0.08			1.36, 0.05	2.04, 0.06	3.40, 0.08		
	0.15, 0.07	0.14, 0.06	0.14, 0.06			0.20, 0.10	0.18, 0.09	0.18, 0.08		
d_2	4.22, 0.10	6.33, 0.12	10.55, 0.15			5.16, 0.10	7.73, 0.12	12.89, 0.15		
	0.20, 0.09	0.21, 0.09	0.26, 0.11			0.25, 0.12	0.26, 0.12	0.33, 0.15		
d_3	5.93, 0.12	8.89, 0.14	**14.82, 0.16**			7.24, 0.12	10.86, 0.14	**18.10, 0.16**		
	0.23, 0.10	0.25, 0.11	**0.55, 0.30**			0.29, 0.13	0.31, 0.14	**0.65, 0.38**		
d_4	6.86, 0.13	10.29, 0.15	17.15, 0.18			8.38, 0.13	12.57, 0.15	20.95, 0.18		
	0.25, 0.10	0.28, 0.12	0.15, 0.05			0.30, 0.14	0.35, 0.16	0.22, 0.09		
d_5	7.38, 0.40	11.06, 0.46	18.44, 0.50			9.01, 0.40	13.51, 0.46	22.52, 0.50		
	0.25, −1.17	0.30, −1.16	0.10, −1.27			0.32, −1.13	0.36, −1.12	0.15, −1.25		

Table S6. (continued)

	$M=0$					$M=1$				
	s_1	s_2	s_3	s_4	s_5	s_1	s_2	s_3	s_4	s_5
					Scenario 5					
d_1	1.15, 0.10	5.73, 0.16	11.45, 0.43	14.31, 0.50		1.40, 0.10	6.99, 0.16	13.99, 0.43	17.48, 0.50	
	0.18, 0.08	0.31, 0.14	0.12, −1.26	0.12, −1.28		0.23, 0.11	0.38, 0.18	0.15, −1.24	0.15, −1.26	
d_2	1.97, 0.14	**9.86, 0.18**	19.71, 0.55	24.64, 0.60		2.41, 0.14	**12.04, 0.18**	24.08, 0.55	30.10, 0.60	
	0.22, 0.09	**0.61, 0.34**	0.62, −0.97	0.75, −0.86		0.28, 0.12	**0.68, 0.39**	0.69, −0.91	0.80, −0.80	
d_3	2.00, 0.48	10.00, 0.55	19.99, 0.65	24.99, 0.70		2.44, 0.48	12.21, 0.55	24.42, 0.65	30.52, 0.70	
	0.22, −1.21	0.62, −0.97	0.64, −0.98	0.77, −0.87		0.28, −1.18	0.69, −0.91	0.70, −0.92	0.82, −0.81	
d_4	2.00, 0.55	10.00, 0.60	20.00, 0.68	25.00, 0.72		2.44, 0.55	12.21, 0.60	24.43, 0.68	30.53, 0.72	
	0.22, −1.23	0.62, −0.98	0.64, −0.99	0.77, −0.88		0.28, −1.20	0.69, −0.92	0.70, −0.93	0.82, −0.82	
					Scenario 6					
d_1	0.28, 0.05	2.09, 0.06	2.79, 0.08	3.48, 0.14		0.34, 0.05	2.55, 0.06	3.40, 0.08	4.25, 0.14	
	0.12, 0.05	0.14, 0.06	0.23, 0.11	0.23, 0.09		0.15, 0.07	0.18, 0.09	0.29, 0.15	0.29, 0.13	
d_2	1.06, 0.08	7.91, 0.10	10.55, 0.12	13.19, 0.38		1.29, 0.08	9.67, 0.10	12.89, 0.12	16.11, 0.38	
	0.13, 0.05	0.23, 0.10	0.40, 0.20	0.44, −1.04		0.16, 0.07	0.29, 0.14	0.47, 0.25	0.52, −0.99	
d_3	1.48, 0.10	11.11, 0.12	**14.82, 0.14**	18.52, 0.48		1.81, 0.10	13.57, 0.12	**18.10, 0.14**	22.62, 0.48	
	0.13, 0.05	0.29, 0.13	**0.63, 0.40**	0.58, −0.98		0.17, 0.07	0.35, 0.18	**0.72, 0.47**	0.65, −0.92	
d_4	1.72, 0.55	12.87, 0.60	17.15, 0.68	21.44, 0.72		2.10, 0.55	15.71, 0.60	20.95, 0.68	26.19, 0.72	
	0.14, −1.29	0.33, −1.19	0.15, −1.30	0.64, −1.00		0.17, −1.26	0.40, −1.14	0.18, −1.27	0.71, −0.94	
					Scenario 7					
d_1	1.15, 0.10	5.73, 0.16	8.59, 0.43	10.31, 0.50	12.60, 0.55	1.40, 0.10	6.99, 0.16	10.49, 0.43	12.59, 0.50	15.39, 0.55
	0.18, 0.08	0.31, 0.14	0.12, −1.26	0.12, −1.28	0.10, −1.31	0.23, 0.11	0.38, 0.18	0.15, −1.24	0.15, −1.26	0.13, −1.29
d_2	1.97, 0.14	**9.86, 0.18**	14.79, 0.55	17.74, 0.60	21.69, 0.65	2.41, 0.14	**12.04, 0.18**	18.06, 0.55	21.67, 0.60	26.49, 0.65
	0.22, 0.09	**0.61, 0.34**	0.46, −1.08	0.56, −1.03	0.63, −0.99	0.28, 0.12	**0.68, 0.39**	0.54, −1.03	0.63, −0.97	0.70, −0.93
d_3	2.00, 0.48	10.00, 0.55	14.99, 0.65	17.99, 0.70	21.99, 0.74	2.44, 0.48	12.21, 0.55	18.31, 0.65	21.97, 0.70	26.86, 0.74
	0.22, −1.21	0.62, −0.97	0.48, −1.10	0.58, −1.04	0.65, −1.00	0.28, −1.18	0.69, −0.91	0.56, −1.05	0.65, −0.99	0.71, −0.94
d_4	2.00, 0.55	10.00, 0.60	15.00, 0.68	18.00, 0.72	22.00, 0.77	2.44, 0.55	12.21, 0.60	18.32, 0.68	21.98, 0.72	26.87, 0.77
	0.22, −1.23	0.62, −0.98	0.48, −1.11	0.58, −1.05	0.65, −1.01	0.28, −1.20	0.69, −0.92	0.56, −1.05	0.65, −0.99	0.72, −0.95
					Scenario 8					
d_1	0.28, 0.05	2.09, 0.06	2.79, 0.08	3.06, 0.14	3.34, 0.36	0.34, 0.05	2.55, 0.06	3.40, 0.08	3.74, 0.14	4.08, 0.36
	0.12, 0.05	0.14, 0.06	0.23, 0.11	0.14, 0.04	0.12, −1.24	0.15, 0.07	0.18, 0.09	0.29, 0.15	0.18, 0.06	0.15, −1.22
d_2	1.06, 0.08	7.91, 0.10	10.55, 0.12	11.61, 0.38	12.66, 0.49	1.29, 0.08	9.67, 0.10	12.89, 0.12	14.18, 0.38	15.47, 0.49
	0.13, 0.05	0.23, 0.10	0.40, 0.20	0.28, −1.15	0.26, −1.20	0.16, 0.07	0.29, 0.14	0.47, 0.25	0.34, −1.11	0.32, −1.16
d_3	1.48, 0.10	11.11, 0.12	**14.82, 0.14**	16.30, 0.48	17.78, 0.55	1.81, 0.10	13.57, 0.12	**18.10, 0.14**	19.90, 0.48	21.71, 0.55
	0.13, 0.05	0.29, 0.13	**0.63, 0.40**	0.38, −1.11	0.36, −1.15	0.17, 0.07	0.35, 0.18	**0.72, 0.47**	0.46, −1.07	0.44, −1.10
d_4	1.72, 0.55	12.87, 0.60	17.15, 0.68	18.87, 0.72	20.59, 0.76	2.10, 0.55	15.71, 0.60	20.95, 0.68	23.05, 0.72	25.14, 0.76
	0.14, −1.29	0.33, −1.19	0.15, −1.30	0.45, −1.14	0.43, −1.17	0.17, −1.26	0.40, −1.14	0.18, −1.27	0.52, −1.09	0.51, −1.11
					Scenario 9					
d_1	1.4, 0.4	2.8, 0.4	3.1, 0.4			1.7, 0.4	3.4, 0.4	3.7, 0.4		
	0.2, −1.2	0.12, −1.2	0.13, −1.2			0.21, −1.2	0.14, −1.2	0.15, −1.2		
d_2	5.3, 0.48	10.6, 0.5	11.6, 0.53			6.4, 0.48	12.9, 0.5	14.2, 0.53		
	0.27, −1.15	0.23, −1.18	0.26, −1.17			0.28, −1.14	0.25, −1.17	0.28, −1.15		
d_3	7.4, 0.5	14.8, 0.62	16.3, 0.64			9.0, 0.5	18.1, 0.62	19.9, 0.64		
	0.31, −1.12	0.31, −1.15	0.36, −1.1			0.32, −1.11	0.36, −1.12	0.67, −0.83		

Table S6.(continued)

	$M=0$					$M=1$				
	s_1	s_2	s_3	s_4	s_5	s_1	s_2	s_3	s_4	s_5
Scenario 10										
d_1	$1.1, 0.4$<br>$0.1, -1.2$	$5.7, 0.5$<br>$0.1, -1.3$	$11.5, 0.4$<br>$0.1, -1.2$			$1.4, 0.4$<br>$0.2, -1.2$	$7.0, 0.5$<br>$0.2, -1.2$	$14.0, 0.4$<br>$0.2, -1.2$		
d_2	$2.0, 0.4$<br>$0.2, -1.2$	$9.9, 0.5$<br>$0.2, -1.2$	$19.7, 0.6$<br>$0.3, -1.2$			$2.4, 0.4$<br>$0.2, -1.2$	$12.0, 0.5$<br>$0.3, -1.2$	$24.1, 0.6$<br>$0.3, -1.2$		
d_3	$2.0, 0.5$<br>$0.2, -1.3$	$10.0, 0.6$<br>$0.2, -1.2$	$20.0, 0.7$<br>$0.3, -1.2$			$2.4, 0.5$<br>$0.2, -1.2$	$12.2, 0.6$<br>$0.3, -1.2$	$24.4, 0.7$<br>$0.3, -1.2$		
d_4	$2.0, 0.6$<br>$0.2, -1.3$	$10.0, 0.6$<br>$0.2, -1.3$	$20.0, 0.7$<br>$0.3, -1.2$			$2.4, 0.6$<br>$0.2, -1.3$	$12.2, 0.6$<br>$0.3, -1.2$	$24.4, 0.7$<br>$0.3, -1.2$		
Scenario 11										
d_1	$1.11, 0.42$<br>$0.12, -1.26$	$1.67, 0.46$<br>$0.14, -1.26$	$2.79, 0.48$<br>$0.14, -1.26$			$1.36, 0.42$<br>$0.15, -1.24$	$2.04, 0.46$<br>$0.18, -1.23$	$3.40, 0.48$<br>$0.18, -1.24$		
d_2	$4.22, 0.50$<br>$0.16, -1.26$	$6.33, 0.52$<br>$0.21, -1.23$	$10.55, 0.55$<br>$0.26, -1.21$			$5.16, 0.50$<br>$0.20, -1.23$	$7.73, 0.52$<br>$0.26, -1.20$	$12.89, 0.55$<br>$0.33, -1.17$		
d_3	$5.93, 0.52$<br>$0.18, -1.25$	$8.89, 0.54$<br>$0.25, -1.21$	$14.82, 0.56$<br>$0.55, -1.02$			$7.24, 0.52$<br>$0.23, -1.22$	$10.86, 0.54$<br>$0.31, -1.18$	$18.10, 0.56$<br>$0.65, -0.94$		
d_4	$6.86, 0.53$<br>$0.19, -1.25$	$10.29, 0.55$<br>$0.28, -1.20$	$17.15, 0.58$<br>$0.15, -1.27$			$8.38, 0.53$<br>$0.25, -1.22$	$12.57, 0.55$<br>$0.35, -1.16$	$20.95, 0.58$<br>$0.22, -1.23$		
d_5	$7.38, 0.60$<br>$0.20, -1.26$	$11.06, 0.66$<br>$0.30, -1.22$	$18.44, 0.70$<br>$0.10, -1.33$			$9.01, 0.60$<br>$0.25, -1.23$	$13.51, 0.66$<br>$0.36, -1.18$	$22.52, 0.70$<br>$0.15, -1.31$		
Scenario 12										
d_1	$1.1, 0.4$<br>$0.1, -1.3$	$5.7, 0.5$<br>$0.1, -1.3$	$8.6, 0.5$<br>$0.1, -1.3$	$11.5, 0.5$<br>$0.1, -1.3$		$1.4, 0.4$<br>$0.1, -1.3$	$7.0, 0.5$<br>$0.1, -1.3$	$10.5, 0.5$<br>$0.2, -1.3$	$14.0, 0.5$<br>$0.2, -1.3$	
d_2	$2.0, 0.4$<br>$0.1, -1.3$	$9.9, 0.5$<br>$0.1, -1.3$	$14.8, 0.6$<br>$0.2, -1.2$	$19.7, 0.6$<br>$0.2, -1.2$		$2.4, 0.4$<br>$0.1, -1.3$	$12.0, 0.5$<br>$0.2, -1.2$	$18.1, 0.6$<br>$0.3, -1.2$	$24.1, 0.6$<br>$0.3, -1.2$	
d_3	$2.0, 0.5$<br>$0.1, -1.3$	$10.0, 0.6$<br>$0.1, -1.3$	$15.0, 0.7$<br>$0.2, -1.3$	$20.0, 0.7$<br>$0.2, -1.3$		$2.4, 0.5$<br>$0.1, -1.3$	$12.2, 0.6$<br>$0.2, -1.3$	$18.3, 0.7$<br>$0.3, -1.2$	$24.4, 0.7$<br>$0.3, -1.2$	
d_4	$2.0, 0.6$<br>$0.1, -1.3$	$10.0, 0.6$<br>$0.1, -1.3$	$15.0, 0.7$<br>$0.2, -1.3$	$20.0, 0.7$<br>$0.2, -1.3$		$2.4, 0.6$<br>$0.1, -1.3$	$12.2, 0.6$<br>$0.2, -1.3$	$18.3, 0.7$<br>$0.3, -1.3$	$24.4, 0.7$<br>$0.3, -1.2$	
Scenario 13										
d_1	$1.1, 0.4$<br>$0.1, -1.3$	$5.7, 0.5$<br>$0.1, -1.3$	$8.6, 0.5$<br>$0.1, -1.3$	$10.3, 0.5$<br>$0.1, -1.3$	$12.6, 0.6$<br>$0.1, -1.3$	$1.4, 0.4$<br>$0.2, -1.2$	$7.0, 0.5$<br>$0.2, -1.2$	$10.5, 0.5$<br>$0.1, -1.3$	$12.6, 0.5$<br>$0.1, -1.3$	$15.4, 0.6$<br>$0.1, -1.3$
d_2	$2.0, 0.4$<br>$0.1, -1.3$	$9.9, 0.5$<br>$0.2, -1.2$	$14.8, 0.6$<br>$0.2, -1.3$	$17.7, 0.6$<br>$0.2, -1.3$	$21.7, 0.7$<br>$0.2, -1.3$	$2.4, 0.4$<br>$0.2, -1.2$	$12.0, 0.5$<br>$0.2, -1.2$	$18.1, 0.6$<br>$0.2, -1.2$	$21.7, 0.6$<br>$0.2, -1.2$	$26.5, 0.7$<br>$0.3, -1.2$
d_3	$2.0, 0.5$<br>$0.1, -1.3$	$10.0, 0.6$<br>$0.2, -1.3$	$15.0, 0.7$<br>$0.2, -1.3$	$18.0, 0.7$<br>$0.2, -1.3$	$22.0, 0.7$<br>$0.2, -1.3$	$2.4, 0.5$<br>$0.2, -1.2$	$12.2, 0.6$<br>$0.2, -1.2$	$18.3, 0.7$<br>$0.2, -1.3$	$22.0, 0.7$<br>$0.2, -1.3$	$26.9, 0.7$<br>$0.3, -1.3$
d_4	$2.0, 0.6$<br>$0.1, -1.3$	$10.0, 0.6$<br>$0.2, -1.3$	$15.0, 0.7$<br>$0.2, -1.3$	$18.0, 0.7$<br>$0.2, -1.3$	$22.0, 0.8$<br>$0.2, -1.3$	$2.4, 0.6$<br>$0.2, -1.3$	$12.2, 0.6$<br>$0.2, -1.3$	$18.3, 0.7$<br>$0.2, -1.3$	$22.0, 0.7$<br>$0.2, -1.3$	$26.9, 0.8$<br>$0.3, -1.3$

Table S7. Summary of simulation results for scenarios in Table S6: percentage of correct selection (PCS) of the optimal dose-schedule, percentage of no selection due to early termination or failed final selection criteria (empty $\mathcal{A}_{M,n}$) and average sample size, for the proposed design.

Scenario	$M = 0$			$M = 1$		
	PCS(# Pts)	No Sel%	Total # Pts	PCS(# of Pts)	No Sel%	Total # Pts
1	78.5(15.2)	1.4	60.0	76.1(12.5)	0.1	59.8
2	45.1(9.7)	2.4	59.4	47.1(8.6)	0.5	59.7
3	65.9(13.7)	3.7	58.9	63.7(11.0)	0.8	59.9
4	54.1(8.9)	14.0	55.9	57.4(6.6)	4.6	59.1
5	83.0(17.0)	2.2	59.6	81.5(13.0)	0.8	59.7
6	45.9(7.1)	1.0	59.7	47.2(5.7)	0.4	59.7
7	80.0(14.2)	2.3	59.2	77.5(10.4)	0.7	60.0
8	40.8(5.9)	4.7	58.9	41.9(4.4)	0.8	59.8
9		97.7	24.7		90.0	32.9
10		97.3	24.9		90.2	33.1
11		96.9	28.6		90.8	33.5
12		99.8	24.0		95.7	31.8
13		98.5	29.8		90.3	38.8

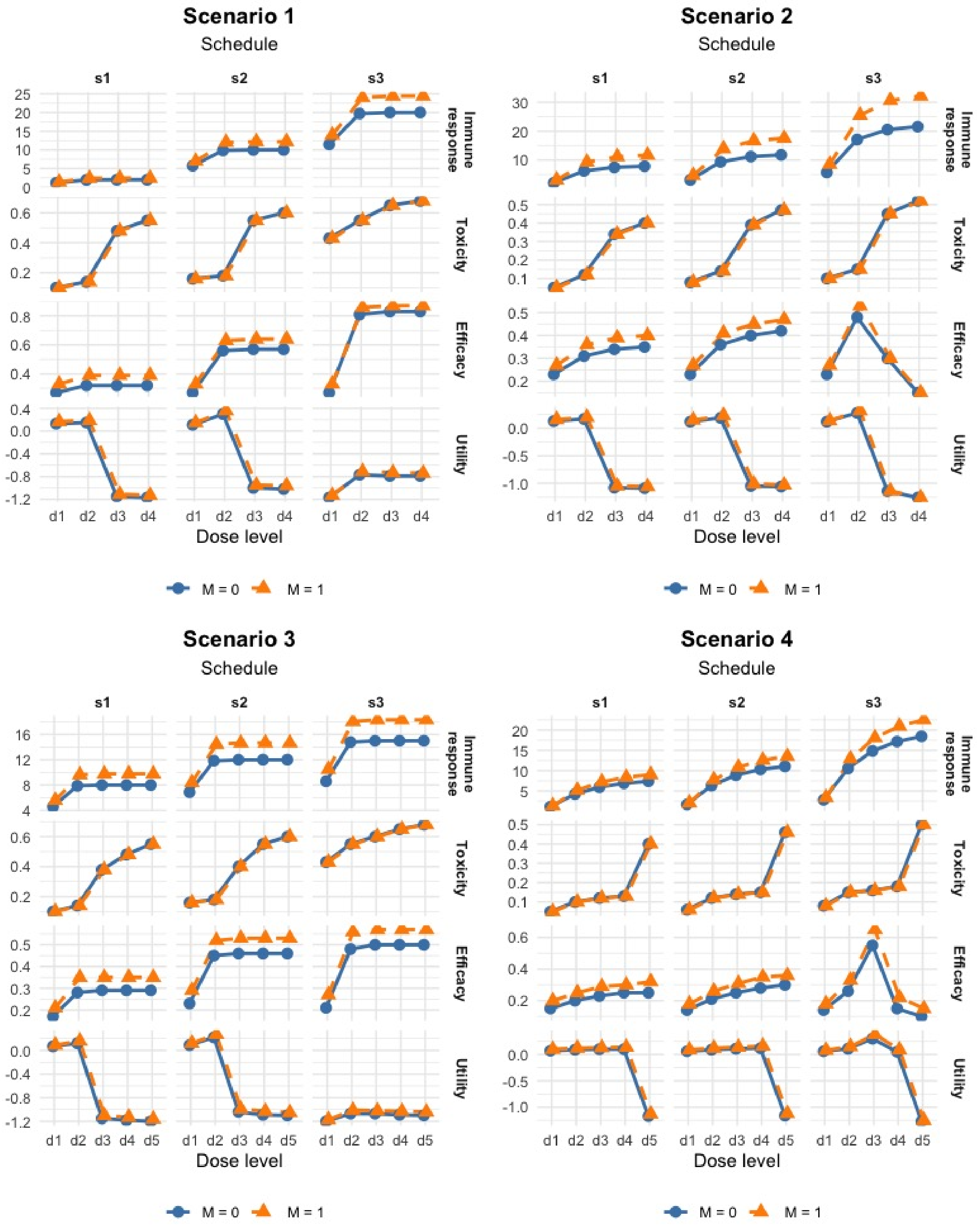

Fig S1: Dose-response curves for the scenarios 1-4 in Table S6.

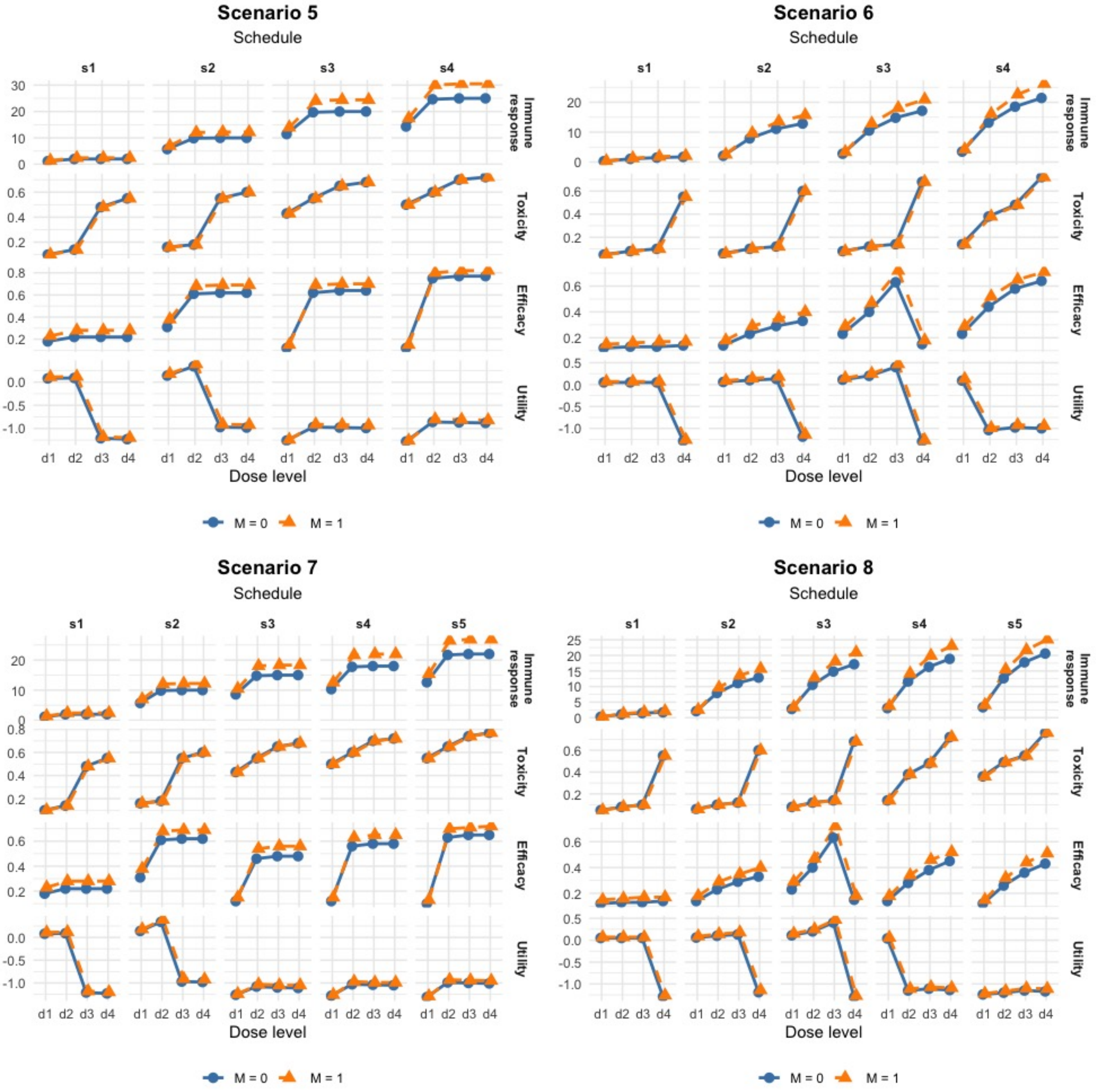

Fig S2: Dose-response curves for the scenarios 5-8 in Table S6.

Random Scenarios

In addition to the simulation study based on fixed scenarios, a second simulation study was performed using randomly generated dose–schedule–toxicity, immune-response, and efficacy surfaces to evaluate the general robustness of the proposed design. Following a non-parametric random-scenario generation algorithm that preserves monotonicity of toxicity and immune response within each schedule, random scenarios were generated for each combination of subgroup-specific optimal dose–schedule pairs.

1. **Specify design targets.**

   The target toxicity threshold was set at $\phi_T = 0.30$ and the target efficacy threshold at $\phi_E = 0.30$. The prespecified optimal dose–schedule combinations were defined as (j_0^*, k_0^*) for the biomarker-negative subgroup ($M = 0$) and (j_1^*, k_1^*) for the biomarker-positive subgroup ($M = 1$).

2. **Generate toxicity probabilities.**

   For each schedule $k = 1, \ldots, K$:

   (a) If k contained either subgroup's prespecified optimum, the minimum of the highest safe dose j^* was identified. The toxicity probabilities for doses $1, \ldots, j^*$ were sampled from Uniform$(0, \phi_T - 0.1)$ and sorted in increasing order, whereas those for doses $j^*+1, ..., J$ were drawn from Uniform$(p(j^*, k), 1)$.

   (b) For schedules without an optimal dose, all $p_{j,k}$ were sampled from Uniform$(0, 1)$ and sorted.

3. **Generate immune-response means $\mu_Z((j, k), M)$.**

   For each subgroup M and schedule k, the maximal immune level $\mu_Z((J, k), M)$ was drawn from Uniform$(u_{\min}, u_{\max})$. The baseline immune level $\mu_Z((1, k), M)$ was drawn

as Uniform$(0.05, 0.20) \times \mu_Z((J,k),M)$. A plateau index j^{plt} was uniformly chosen from $\{1,\dots,J\}$, and the plateau height $\mu_Z((j^{\text{plt}},k),M) \in [0.9\mu_Z((J,k),M), \mu_Z((J,k),M)]$ was selected. Immune means up to the plateau were generated as a monotone-increasing sequence, followed by a constant plateau thereafter.

For the biomarker-positive subgroup ($M = 1$), a biomarker effect was introduced as

$$\mu_Z((j,k),1) = \lambda_k \, \mu_Z((j,k),0) + \epsilon_{j,k}, \quad \lambda_k \sim \text{Uniform}(1.1, 1.4), \;\; \epsilon_{j,k} \sim N(0, \sigma_\mu^2),$$

with sorting applied to ensure monotonicity.

4. **Generate efficacy probabilities** $q_{2,(j,k),M}$**.**

   Each efficacy surface $q_{2,(j,k),M}$ was linked to the corresponding immune profile. The plateau efficacy $q_{2,(j^{\text{plt}},k),M}$ was sampled from Uniform$(0.20, 0.60)$ for $M = 0$, and scaled for $M = 1$ by

$$q_{2,(j^{\text{plt}},k),1} = \rho_k^{(2)} \, q_{2,(j^{\text{plt}},k),0}, \quad \rho_k^{(2)} \sim \text{Uniform}(1.05, 1.25).$$

   Lower doses followed a smooth monotone increase up to the plateau.

5. **Generate total response probabilities** $\pi_{(j,k),M}$**.**

   For each (k,M), the upper bound $q_U^{(M)}$ and curve shape were generated based on whether schedule k contained the subgroup-specific optimal dose. If k contained optimal dose, the upper bound was drawn from Uniform$(\phi_E + 0.1, 1)$; otherwise, from Uniform$(0,1)$. The indicator of a plateau-shaped curve was defined as $I(U < 0.5)$ with $U \sim$ Uniform$(0,1)$.

   If $I = 1$, $\pi_{(j,k),M}$ followed a plateau pattern: probabilities for $j = 1,\dots,j^{\text{plt}}$ were sampled as a sorted vector from Uniform$(0, q_U^{(M)})$, and for $j > j^{\text{plt}}$, $\pi_{(j,k),M}$ was fixed

at $\pi_{(j^{\text{plt}},k),M}$. If $I = 0$, a bell-shaped curve was generated by selecting a most effective dose $j^{\text{MED}} \sim \text{Uniform}(j^{\text{plt}}, J)$, assigning $\pi_{(j^{\text{MED}},k),M}$ as the maximum of J draws from $\text{Uniform}(0, q_U^{(M)})$, and filling values on either side with increasing and decreasing sorted vectors, respectively.

All $\pi_{(j,k),M}$ were constrained such that $\pi_{(j,k),M} \geq q_{2,(j,k),M}$, truncated to $[0, 1]$, and adjusted to ensure $\pi_{(j,k),1} \geq \pi_{(j,k),0}$.

6. **Determine admissible sets and subgroup-specific optimal dose–schedule combinations.**

   A combination (j, k) was defined as admissible for subgroup M if $p_{j,k} < \phi_T$ and $\pi_{(j,k),M} > \phi_E$. The utility was calculated, and the algorithm iteratively looped through Steps 2–5 until each subgroup's prespecified (j_M^*, k_M^*) lay within the admissible set and maximized utility.

Through this non-parametric generation procedure, random scenarios were simulated for each subgroup-specific configuration, ensuring biological meaningful dose-response relationship within each schedule , and biomarker-specific optimal dose–schedules that were both admissible and utility-maximizing.

Due to computational constraints, we randomly generated 20 scenarios for each prespecified true optimal dose–schedule combination, (d_2, s_1) and (d_2, s_2), for both subgroups respectively. Under each scenario, we conducted 2,000 simulated trials. The resulting operating characteristics are summarized in Figures S3 and S4.

Fig S3: Summary of simulation results of random scenarios with true optimal dose-schedule combination (d_2, s_1).

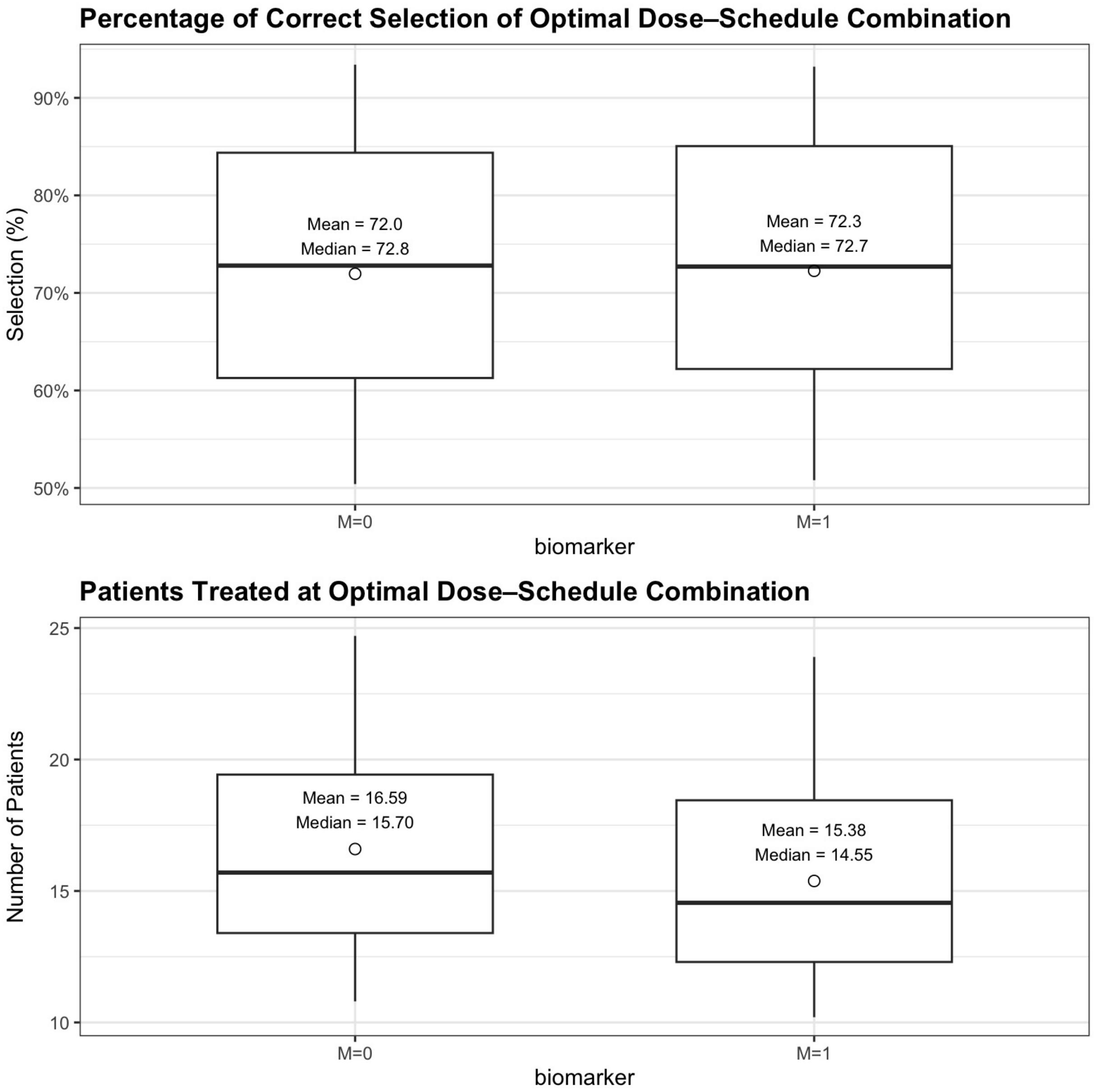

Fig S4: Summary of simulation results of random scenarios with true optimal dose-schedule combination (d_2, s_2).

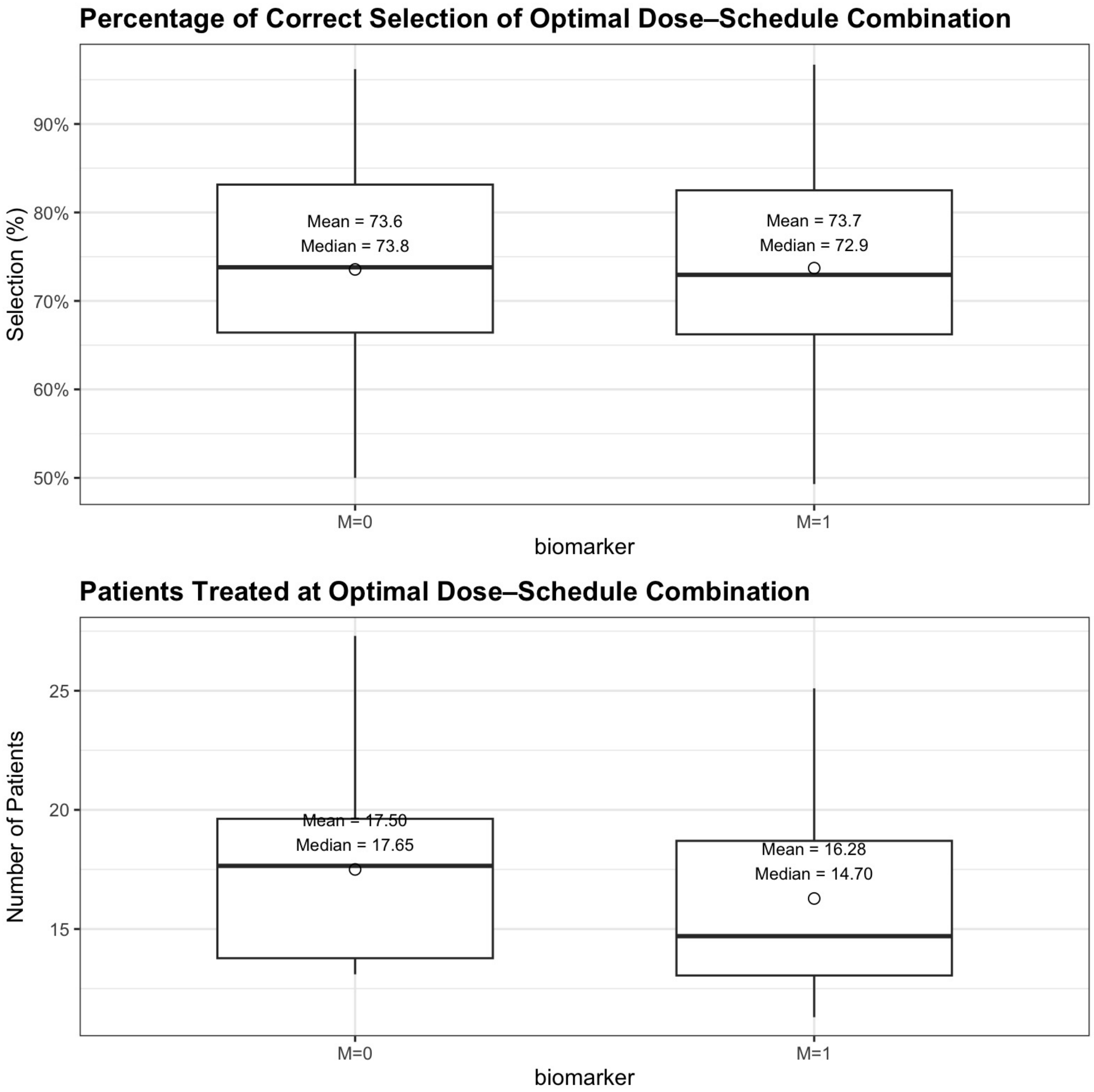

R Implementation for Simulation Studies and Real Trial Conduct

This section provides the `R` implementation for both the simulation studies and the real trial conduct of the proposed two-stage Bayesian biomarker-guided dose–schedule optimization design. The accompanying code includes example datasets, simulation functions, and decision-making functions for Stage II interim and final analyses.

Simulation Study

All simulations were conducted in `R` (version 4.3.0). The proposed Bayesian biomarker-guided dose–schedule phase I/II design was implemented through a custom simulation function, `get.oc.dose.schedule()`, which generates multiple independent trials under user-specified true toxicity, efficacy, and biomarker-dependent outcome probabilities. The following section provides documentation and a fully reproducible example.

Function: `get.oc.dose.schedule()`

Purpose: To evaluate operating characteristics (OCs) of the proposed biomarker-guided Bayesian dose–schedule design under specified true toxicity and efficacy profiles for each biomarker subgroup.

Arguments:

- `J`, `K`: Number of dose levels (J) and schedules (K).
- `cohortsize`: Number of patients per cohort (e.g., 3).
- `ncohort`: Total number of cohorts per simulated trial.

- `toxprob`: $J \times K$ matrix of true toxicity probabilities for each dose–schedule combination.
- `mu0`, `mu1`: $J \times K$ matrix of true mean parameters for the immune response for biomarker-negative ($M = 0$) and biomarker-positive ($M = 1$) subgroups.
- `p0Y0`, `p0Y1`, `p0Y2`: $J \times K$ matrix of true probabilities of efficacy outcomes (e.g., progressive disease (PD), stable disease (SD) and complete remission or partial remission (CR/PR)) for the biomarker-negative subgroup.
- `p1Y0`, `p1Y1`, `p1Y2`: $J \times K$ matrix of true probabilities of efficacy outcomes (e.g., progressive disease (PD), stable disease (SD) and complete remission or partial remission (CR/PR)) for the biomarker-negative subgroup.
- `phiT`: Toxicity upper bound defining admissibility (e.g., 0.3).
- `cutoffT_I`: Posterior cutoff for stage I toxicity monitoring.
- `cutoffT`: Posterior cutoff for stage II toxicity admissibility.
- `phiE`: Lower efficacy bound defining clinical relevance (e.g., 0.3).
- `cutoffE`: Posterior cutoff for efficacy admissibility.
- `ntrial`: Number of simulated trials (e.g., 1000).

Output: The function returns a list containing:

- `sim_N`: A 3D array ($J \times K \times ntrial$) of patient allocations across all dose–schedule combinations.
- `sim_N0`, `sim_N1`: 3D arrays of patient allocations for biomarker subgroups $M = 0$ and $M = 1$.
- `counts0`, `counts1`: Vectors of selected optimal dose–schedule indices for each subgroup.

Example Usage: scenario 1 in Table 1 from main simulation study

```
toxprob <- matrix(c(0.10,0.16,0.43,
                    0.14,0.18,0.55,
                    0.48,0.55,0.65), nrow = 3, byrow = TRUE)

mu0 <- matrix(c(3.623718, 7.247437, 7.972181,
                8.946008, 17.892016, 19.681217,
                9.825776, 19.651553, 21.616708), nrow = 3, byrow = TRUE)

mu1 <- matrix(c(4.42602, 8.85204, 9.737243,
                10.92668, 21.85336, 24.038693,
                12.00123, 24.00246, 26.402706), nrow = 3, byrow = TRUE)

p0Y0 <- matrix(c(0.5498340,0.5498340,0.5498340,
                 0.4176984,0.2964088,0.2747004,
                 0.3964675,0.2610706,0.2378591), nrow = 3, byrow = TRUE)
p0Y1 <- matrix(c(0.3504155,0.3504155,0.3504155,
                 0.4235800,0.4604514,0.4620402,
                 0.4327079,0.4619688,0.4596674), nrow = 3, byrow = TRUE)
p0Y2 <- matrix(c(0.09975049,0.09975049,0.09975049,
                 0.15872158,0.24313977,0.26325933,
                 0.17082463,0.27696060,0.30247340), nrow = 3, byrow = TRUE)

p1Y0 <- matrix(c(0.4750208,0.4750208,0.4750208,
                 0.3470053,0.2378582,0.2191026,
```

```
                 0.3273477,0.2074424,0.1877872), nrow = 3, byrow = TRUE)
p1Y1 <- matrix(c(0.3948707,0.3948707,0.3948707,
                 0.4500145,0.4596673,0.4555043,
                 0.4550671,0.4517255,0.4429888), nrow = 3, byrow = TRUE)
p1Y2 <- matrix(c(0.1301085,0.1301085,0.1301085,
                 0.2029802,0.3024745,0.3253931,
                 0.2175853,0.3408321,0.3692240), nrow = 3, byrow = TRUE)

result <- get.oc.dose.schedule(
  cohortsize = 3, ncohort = 40,
  toxprob = toxprob,
  mu0 = mu0, mu1 = mu1,
  p0Y0 = p0Y0, p0Y1 = p0Y1, p0Y2 = p0Y2,
  p1Y0 = p1Y0, p1Y1 = p1Y1, p1Y2 = p1Y2,
  J = 3, K = 3,
  phiT = 0.3, cutoffT_I = 0.05, cutoffT = 0.2,
  phiE = 0.3, cutoffE = 0.2, ntrial = 2000
)

# Summarize operating characteristics
apply(result$sim_N, c(1,2), mean)        # Avg. patient allocation
apply(result$sim_N0, c(1,2), mean)       # Biomarker-negative
apply(result$sim_N1, c(1,2), mean)       # Biomarker-positive

round(table(result$counts0) / sum(table(result$counts0)) * 100, 2)
round(table(result$counts1) / sum(table(result$counts1)) * 100, 2)
```

Sample Output:

```
> round(table(result$counts0)/sum(table(result$counts0))*100,2)
# k
# j      1    2    3
# 1  6.1  6.0  0.6
# 2 22.6 61.9  0.4
# 3  1.7  0.7  0.0

> round(table(result$counts1)/sum(table(result$counts1))*100,2)
# k
# j      1    2    3
# 1  7.0  6.2  0.7
# 2 23.0 60.1  0.4
# 3  2.1  0.5  0.0

> apply(result$sim_N0, c(1,2), mean)
#       [,1]   [,2]  [,3]
# [1,] 10.718 10.986 7.661
# [2,] 11.536 11.575 2.093
# [3,]  2.518  1.924 1.120

> apply(result$sim_N1, c(1,2), mean)
#       [,1]   [,2]  [,3]
# [1,] 11.015 10.984 7.716
# [2,] 11.319 11.192 2.080
```

```
# [3,]  2.432  1.932 1.199
```

```
> apply(result$sim_N, c(1,2), mean)
#       [,1]   [,2]   [,3]
# [1,] 21.733 21.970 15.377
# [2,] 22.855 22.767  4.173
# [3,]  4.950  3.856  2.319
```

The `apply()` commands calculate average patient allocation across dose–schedule combinations, while the `table()` functions summarize the proportion of simulated trials selecting each optimal dose–schedule for the biomarker-defined subgroups. All R scripts and implementation instructions are available at the accompanying GitHub repository.

Stage II Interim and Final Analysis Example

Stage I of the proposed design adopts a standard Beta–Binomial model for dose-schedule exploration based on toxicity monitoring. The following examples illustrate the implementation of the interim and final analyses for Stage II using the provided `R` functions.

Interim Analysis. The interim decision can be obtained using the function `run.real.trial.interim()` which updates posterior estimates based on the currently available data and determines the admissible dose–schedule combinations for each biomarker subgroup.

```
# Run interim decision
interim_result <- run.real.trial.interim(
    data_obs = data_obs,
    J = 3, K = 3,
    phiE = 0.3, phiT = 0.3,
```

```
    cutT = 0.2, cutE = 0.2
)
```

A representative output is shown below:

```
================= Interim Analysis Summary =================
Termination Decision:  No
→ Continue both M0 and M1.
Recommended next (M0): (j = 2 , k = 1 )
Recommended next (M1): (j = 2 , k = 1 )
=============================================================
```

This output indicates that both subgroups (`M0` and `M1`) satisfied the admissibility criteria and are recommended to continue enrollment at dose–schedule combination $(j, k) = (2, 1)$.

Final Analysis. At the completion of the trial, the final analysis can be conducted using the function `run.stageII.final()`, which re–estimates posterior probabilities of efficacy and toxicity based on all accumulated data and identifies the optimal dose–schedule combinations for each biomarker subgroup.

```
# Run final decision
final_out <- run.stageII.final(
    data_stageII = stageII_data,
    J = 3, K = 3,
    phiE = 0.3, phiT = 0.3,
    cutT = 0.2, cutE = 0.2
)
```

A representative output is as follows:

```
================= Final Stage II Analysis =================
Termination Decision: No
→ Both subgroups continue.
Optimal for M0: (j = 1 , k = 1)
Optimal for M1: (j = 2 , k = 1)
============================================================
```

This result demonstrates that both biomarker subgroups satisfy the posterior admissibility criteria, with final optimal dose–schedule combinations identified as $(j, k) = (1, 1)$ for subgroup M=0 and $(j, k) = (2, 1)$ for subgroup M=1.

All `R` scripts and detailed implementation instructions are available at the accompanying GitHub repository: `https://github.com/FrankQiu20`, ensuring full reproducibility of the simulation studies and real–trial implementation.

References

Cheung, Y. K. (2014). Simple benchmark for complex dose finding studies. Biometrics, 70(2), 389-397.
